# Two-layer hierarchical MPPT strategy of PV systems based on AF-EPO algorithm under complex irradiance conditions

**DOI:** 10.3389/fnbot.2026.1882865

**Published:** 2026-08-14

**Authors:** Lei Wang, Jinzhao Cui, Chao Ge, Zhixin Lun

**Affiliations:** 1 College of Intelligence and Information Engineering, Tangshan University, Tangshan, China; 2 College of Electrical Engineering, North China University of Science and Technology, Tangshan, China; 3 University Computer Center, Tangshan University, Tangshan, China

**Keywords:** eagle perching optimization, fuzzy inference system, maximum power point tracking (MPPT), partial shading conditions, photovoltaic power generation

## Abstract

Photovoltaic power generation systems exhibit multi-peak power–voltage characteristics under partial shading conditions, severely limiting the effectiveness of conventional maximum power point tracking methods. This paper proposes a two-layer hierarchical MPPT architecture based on an adaptive fuzzy-weighted eagle perching optimization algorithm, designated AF-EPO. Unlike existing fuzzy-metaheuristic MPPT hybrids that apply fuzzy logic as an exogenous regulator to secondary control variables while retaining fixed-parameter core dynamics, AF-EPO embeds the fuzzy inference system directly into the endogenous EPO scaling factor, jointly driven by iteration progress and population diversity, thereby addressing three persistent limitations of metaheuristic MPPT through structural changes absent from existing fuzzy-hybrid formulations: the fixed-parameter exploration–exploitation dilemma, the computational overhead of unconditional global search, and the residual steady-state power oscillation that persists in all population-based methods. A slope sign-reversal detection module first identifies whether the power–voltage curve is unimodal or multi-modal, activating the global search layer only when partial shading is confirmed. In the global layer, a 25-rule Mamdani fuzzy inference system dynamically adjusts the EPO scaling factor according to iteration progress and population diversity, balancing exploration and exploitation throughout the search. A variable-step incremental conductance controller then refines the operating point to suppress steady-state oscillation. Simulations across three scenarios demonstrate that AF-EPO reduces tracking time by 47.3%, power oscillation rate by 74.6%, and energy loss by 38.2% compared with standard EPO. Hardware experiments on a TMS320F28335 DSP platform confirm tracking efficiencies exceeding 98.1%, with a maximum simulation-to-experiment deviation of 1.1%, validating the practical effectiveness of the proposed method.

## 1 Introduction

Solar photovoltaic (PV) power generation has emerged as one of the most critical technologies for achieving global carbon neutrality and sustainable energy development. The practical performance of PV systems is severely constrained by environmental variability, particularly fluctuations in solar irradiance and temperature. Under uniform irradiance, a PV array exhibits a single well-defined maximum on its power–voltage (*P*–*V*) curve. Under partial shading conditions (PSCs), however, bypass diodes activated across shaded modules introduce multiple local peaks into the *P*–*V* characteristic, and only one represents the true global maximum power point (GMPP) (Nunes et al., 2022). Failure to locate the GMPP results in substantial energy losses that accumulate over the operational lifetime of a system (Mhanni and Lagmich, 2024).

Conventional MPPT methods, including perturb-and-observe (P&O) and incremental conductance (INC), are inherently incapable of distinguishing between local and global maxima under PSCs, frequently converging to a local peak with persistent steady-state oscillations (Abouzeid et al., 2024; Rojas-Galván et al., 2024). These limitations have motivated extensive research into swarm intelligence-based MPPT methods. Particle swarm optimization (PSO), gray wolf optimization (GWO), whale optimization algorithm (WOA), salp swarm algorithm (SSA), marine predators algorithm (MPA), and numerous bio-inspired variants have all been applied to MPPT, demonstrating superior global search capability over conventional methods (Chtita et al., 2022; Jamaludin et al., 2025; Yu et al., 2024; Zhang et al., 2024; Zhong et al., 2024). Fuzzy logic hybrids such as fuzzy-GWO and fuzzy-PSO have further improved tracking accuracy by modulating auxiliary control variables (Kumar and Balakrishna, 2024b). Two-layer hierarchical architectures have additionally been proposed for grid-level power management in PV-storage microgrids (Meziane et al., 2025; Wang et al., 2025). A structured comparison of representative existing methods is provided in Table 1.

**Table 1:** Comparative summary of representative MPPT methods highlighting research gaps addressed by the proposed AF-EPO.

Algorithm	Type	Tracking efficiency (%)	Convergence speed	Oscillation behavior	Primary limitation
P&O	Conventional hill-climbing	~98.8 (uniform only)	Fast	Persistent	Cannot handle multi-peak *P*–*V*
PSO	Swarm intelligence	99.1–99.3	Medium	Residual	Fixed core parameters
GWO	Swarm intelligence	99.2–99.4	Medium	Residual	Fixed core parameters
Fuzzy-GWO	Fuzzy-metaheuristic hybrid	~99.5	Medium	Moderate	Exogenous FIS regulation only
WOA-P&O	Two-stage sequential	~98.8	Medium	Local refinement residual	Fixed iteration-count handoff threshold
SSA hybrid	Two-stage sequential	~98.9	Medium	Hill-climbing residual	Fixed power-ratio switching criterion
**AF-EPO**	**Hierarchical hybrid**	**99.4–99.7**	**Fast**	**Near-zero**	Hardware validation at fixed 25 °C only

Bold values indicate the proposed AF-EPO method.

Despite these advances, a systematic analysis reveals three persistent limitations shared by all existing approaches. First, all fuzzy-metaheuristic hybrids apply the fuzzy inference system to an exogenous auxiliary variable—typically the perturbation step size, inertia weight, or velocity coefficient—while retaining fixed parameters in the core position update dynamics, so that the asymptotic convergence floor cannot be reduced regardless of how the auxiliary variable is modulated. Second, population-based global search is activated unconditionally regardless of whether partial shading is actually present, imposing unnecessary computational overhead under uniform irradiance. Third, all population-based methods produce residual steady-state power oscillations because the global optimizer remains active in steady state. No existing fuzzy-hybrid formulation resolves all three limitations simultaneously through structural architectural changes.

To address these shortcomings, this paper proposes a two-layer hierarchical MPPT architecture based on an adaptive fuzzy-weighted eagle perching optimization algorithm (AF-EPO). The principal contributions are as follows. (1) Endogenous fuzzy-adaptive scaling: AF-EPO embeds a 25-rule Mamdani fuzzy inference system directly into the EPO scaling factor *f*^*t*^, driven jointly by iteration progress τ and population diversity *D*, with a formal convergence proof demonstrating that the mean-squared tracking error decreases geometrically to a tightening invariant set—a guarantee unavailable in any fixed-parameter or exogenously regulated formulation. (2) Sensor-free multi-modal detection: A *P*–*V* slope sign-reversal counting module discriminates between unimodal and multi-modal operating conditions without additional hardware, activating the global search layer only when partial shading is confirmed. (3) Decoupled two-layer architecture: A variable-step INC controller, seeded by the global layer output, achieves near-zero steady-state oscillation through slope-proportional perturbation, decoupling global escape capability from local tracking precision. Comprehensive MATLAB/Simulink simulation and TMS320F28335 DSP hardware validation across three representative scenarios confirms the practical superiority of the proposed architecture.

## 2 Mathematical modeling of the photovoltaic power generation system

### 2.1 Equivalent circuit model of the photovoltaic cell

The single-diode model (SDM) is the most widely adopted representation of a photovoltaic (PV) cell for engineering simulation due to its favorable balance between physical fidelity and computational simplicity (Rawa et al., 2022). In this model, a PV cell is represented by a current source *I*_*ph*_ in parallel with an ideal diode, a series resistance *R*_*s*_ accounting for ohmic contact and bulk material losses, and a shunt resistance *R*_*sh*_ modeling leakage currents across the p–n junction. The terminal current–voltage relationship is described by Equation 1:


$$\begin{array}{l} I=I_{\mathrm{ph}}-I_{0}\left[\exp\left(\frac{V+IR_{s}}{nV_{T}}\right)-1\right]-\frac{V+IR_{s}}{R_{\mathrm{sh}}} \end{array}$$ (1)

where *I*_0_ is the reverse saturation current of the diode, *n* is the ideality factor, and *V*_*T*_ = *kT*/*q* is the thermal voltage, with *k* being the Boltzmann constant, *T* the absolute cell temperature, and *q* the elementary charge. An improved formulation that adds a small resistance in series with the diode provides more accurate power-loss representation and yields an exact analytical solution via Lambert's *W* function as given in Equation 2 (Li et al., 2023; Rawa et al., 2022):


$$\begin{array}{l} V=\frac{nV_{T}}{R_{s}}W\left(\frac{R_{s}I_{0}}{nV_{T}}\exp\left(\frac{R_{s}\left(I_{\mathrm{ph}}+I_{0}\right)+V_{\mathrm{oc}}}{nV_{T}}\right)\right)-\frac{R_{s}\left(I+I_{0}\right)}{1} \end{array}$$ (2)

The five unknown parameters {*I*_*ph*_, *I*_0_, *n, R*_*s*_, *R*_*sh*_} must be identified from measured *I*–*V* data or manufacturer datasheets. Because the governing equation is implicit and highly non-linear, analytical approaches require successive approximations that sacrifice accuracy, whereas metaheuristic optimizers minimize a root-mean-square-error objective function defined in Equation 3


$$\begin{array}{l} \text{RMSE}=\sqrt{\frac{1}{N}\overset{N}{\underset{j=1}{\sum }}{\left(I_{\text{meas},j}-I_{\text{model},j}\right)}^{2}} \end{array}$$ (3)

over the five-dimensional parameter space (Izci et al., 2024; Kullampalayam Murugaiyan et al., 2024). Comparative studies covering opposition-based learning, weighted leader search, prairie dog optimization, and 10 further algorithms confirm that modern nature-inspired methods achieve RMSE values on the order of 10^−4^ for the standard RTC France solar cell, far surpassing gradient-based techniques in both accuracy and robustness (Çetinbaş, 2024; Zga et al., 2025). Further accuracy gains are available through extended double- and triple-diode formulations whose additional recombination currents capture low-irradiance behavior, albeit at greater computational cost (Almutairi and Shaheen, 2025; Çelik et al., 2025).

In engineering practice, the five-parameter model is scaled from the cell level to modules and arrays by adjusting the series and shunt resistances proportionally to the number of cells connected in series *N*_*s*_ and parallel *N*_*p*_ as given in Equation 4:


$$\begin{array}{l} R_{s,\text{array}}=N_{s}R_{s}/N_{p},\;R_{\mathrm{sh},\text{array}}=N_{s}R_{\mathrm{sh}}/N_{p} \end{array}$$ (4)

The photocurrent and saturation current are corrected for operating temperature *T* and irradiance *G* relative to their standard test condition (STC) values as


$$\begin{array}{lll} I_{\mathrm{ph}} & = & \left(I_{\mathrm{ph},\text{STC}}+K_{I},\mathrm{\Delta T}\right)\frac{G}{G_{\text{STC}}} \end{array}$$

$$\begin{array}{lll} I_{0} & = & I_{0,\text{STC}}{\left(\frac{T}{T_{\text{STC}}}\right)}^{3}\exp\left[\frac{qE_{g}}{\mathrm{nk}}\left(\frac{1}{T_{\text{STC}}}-\frac{1}{T}\right)\right] \end{array}$$ (5)

where *K*_*I*_ is the short-circuit current temperature coefficient and *E*_*g*_ is the band-gap energy of silicon (Khyani et al., 2023).

### 2.2 Characteristics analysis of the photovoltaic array under partial shading

**Figure 1:**
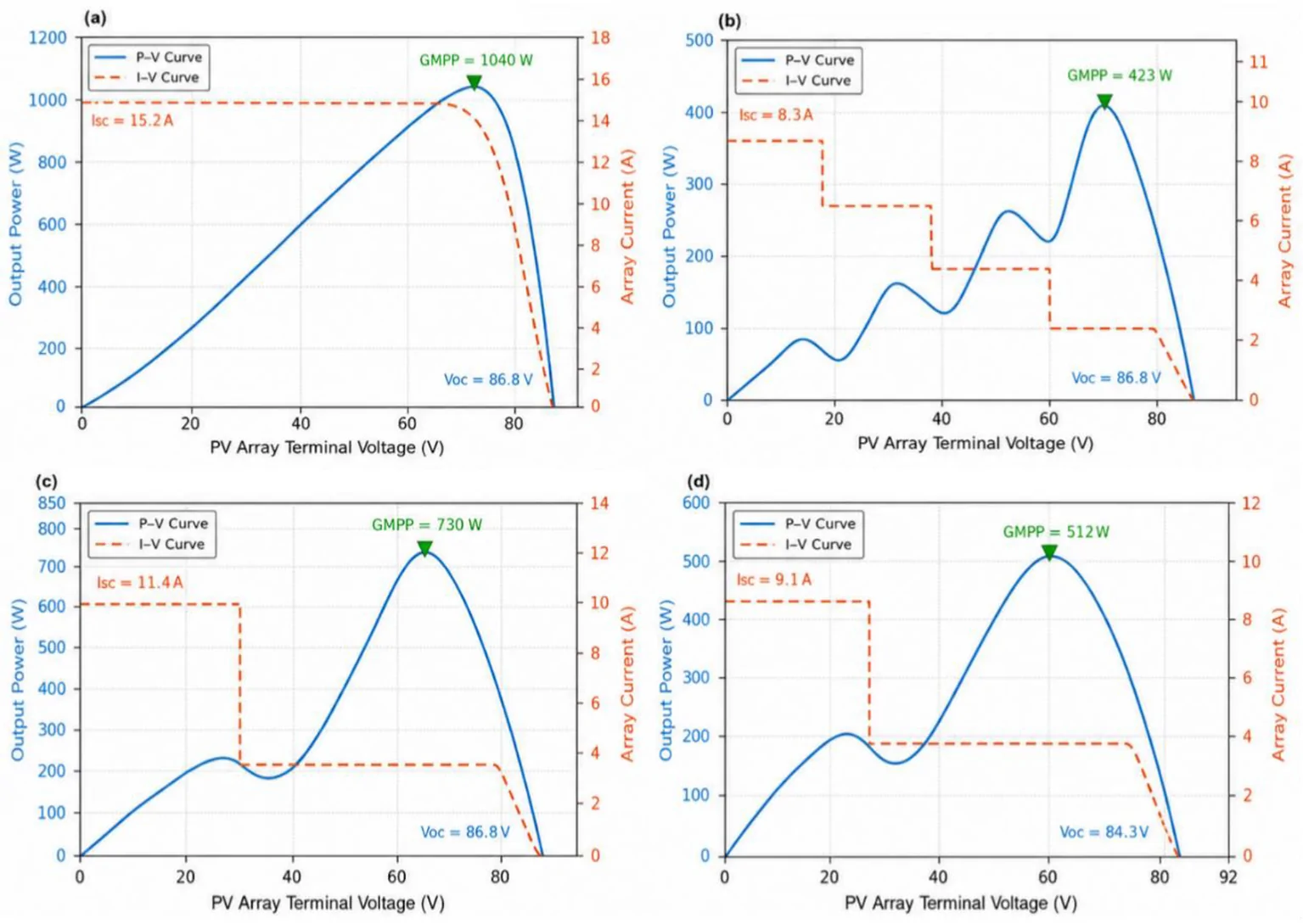
*P*–*V* and *I*–*V* characteristics of a 4 × 4 PV array under different irradiance conditions. **(a)** Uniform irradiance: all strings at 1,000 W/m^2^. **(b)** Static partial shading: strings at 1,000/600/400/200 W/m^2^. **(c)** Dynamic shading phase 1: strings at 1,000/1,000/200/200 W/m^2^. **(d)** Dynamic shading phase 2: strings at 800/800/200/200 W/m^2^.

Under uniform irradiance, the power–voltage (*P*–*V*) curve of a PV array exhibits a single, well-defined global maximum power point (GMPP) satisfying *dP*/*dV* = 0. When partial shading occurs, individual modules in a series string receive different irradiance levels, forcing bypass diodes—connected anti-parallel to each module—to conduct and redirect current around the shaded module (Abad et al., 2025). Each conducting bypass diode introduces a distinct voltage plateau in the current–voltage (*I*–*V*) curve and a corresponding local peak in the *P*–*V* curve, so that a string of *M* modules with *K* distinct irradiance levels produce up to *K* local maxima (Saeed et al., 2022). Simulation of a 4 × 4 array in MATLAB/Simulink across ten interconnection topologies—including series–parallel (SP), total-cross-tied (TCT), bridge-linked (BL), honey-comb (HC), and hybrid variants—demonstrates that TCT consistently yields the highest global maximum power and the lowest mismatch loss Δ*P*_*ml*_ under all tested shading patterns (Saeed et al., 2022). The typical *P*–*V* and *I*–*V* characteristics of the 4 × 4 PV array under uniform irradiance and representative partial shading patterns are illustrated in Figure 1.

The power mismatch loss due to non-uniform irradiance is quantified by Equation 6


$$\begin{array}{l} \Delta P_{\mathrm{ml}}=P_{\text{ideal}}-P_{\text{GMPP}} \end{array}$$ (6)

where *P*_ideal_ is the aggregate power that would be extracted if each module operated independently at its individual MPP. Under dynamic shading scenarios, where shadow boundaries traverse the array over time, the instantaneous positions of all local maxima shift continuously, making static interconnection schemes insufficient to maintain minimum mismatch losses (Shao et al., 2023). Dynamic electrical array reconfiguration (EAR), which periodically switches the interconnection matrix according to measured irradiance distributions, has been shown to reduce daily energy losses by up to 30% compared with fixed SP wiring (Fang and Yang, 2024; Ganesan et al., 2025). The number and relative magnitudes of local maximum power points (LMPPs) depend on the shading pattern, string length, and bypass diode threshold voltage *V*_*D*_≈0.5 − 0.7 V; in the worst case, the GMPP carries only a marginally higher power than the dominant LMPP, making global search indispensable for the MPPT controller (Abad et al., 2025; Bui et al., 2025).

### 2.3 Mathematical model of the boost converter

The boost DC–DC converter serves as the power interface between the PV array and the load or inverter stage, stepping up the array terminal voltage *v*_*PV*_ to a regulated output voltage *v*_*o*_ under continuous conduction mode (CCM) (Khan et al., 2024). Applying state-space averaging over one switching period *T*_*s*_ with duty cycle *d* yields the averaged circuit equations given in Equation 7


$$\begin{array}{lll} L\frac{d\langle i_{L}\rangle }{\mathrm{dt}} & = & \langle v_{\mathrm{PV}}\rangle -\left(1-d\right)\langle v_{o}\rangle \end{array}$$

$$\begin{array}{lll} C\frac{d\langle v_{o}\rangle }{\mathrm{dt}} & = & \left(1-d\right)\langle i_{L}\rangle -\frac{\langle v_{o}\rangle }{R} \end{array}$$ (7)

where *L* is the boost inductance, *C* is the output capacitance, 〈*i*_*L*_〉 is the average inductor current, and *R* is the equivalent load resistance (Hassan et al., 2024; Khan et al., 2024). In steady state, *d*〈*i*_*L*_〉/*dt* = *d*〈*v*_*o*_〉/*dt* = 0, giving the ideal voltage conversion ratio in Equation 8


$$\begin{array}{l} M\left(d\right)=\frac{V_{o}}{V_{\mathrm{PV}}}=\frac{1}{1-d} \end{array}$$ (8)

The duty cycle thus controls the operating voltage of the PV array, and the MPPT algorithm adjusts *d* iteratively so that the array is driven to its GMPP. The instantaneous output power delivered to the load is given by Equation 9


$$\begin{array}{l} P_{o}=\frac{V_{o}^{2}}{R}=\frac{V_{\mathrm{PV}}^{2}}{{\left(1-d\right)}^{2}R} \end{array}$$ (9)

showing that *P*_*o*_ is a monotonically increasing function of *d* for fixed *V*_*PV*_ and *R*, while the PV array power *P*_*PV*_(*d*) = *V*_*PV*_(*d*)·*I*_*PV*_(*d*) has a single peak under uniform irradiance or multiple peaks under partial shading (Hassan et al., 2024). The duty cycle at the maximum power point satisfies Equation 10


$$\begin{array}{l} d^{*}=1-\sqrt{\frac{R_{\mathrm{MPP}}}{R}} \end{array}$$ (10)

where *R*_*MPP*_ = *V*_*MPP*_/*I*_*MPP*_ is the equivalent resistance of the PV array at its MPP (Abdolrasol et al., 2025). For small-signal stability analysis, perturbing around the steady-state operating point $\left(\bar{d},{\bar{V}}_{\mathrm{PV}},{\bar{V}}_{o}\right)$ yields the small-signal transfer function from duty cycle to output voltage given in Equation 11


$$\begin{array}{lll} G_{\mathrm{vd}}\left(s\right) & = & \frac{{\hat{v}}_{o}\left(s\right)}{\hat{d}\left(s\right)}\\ = & \frac{{\bar{V}}_{\mathrm{PV}}}{{\left(1-\bar{d}\right)}^{2}}\cdot \frac{1-\mathrm{sL}{Ī}_{L}/{\left(1-\bar{d}\right)}^{2}{\bar{V}}_{o}}{1+\mathrm{sRC}/\left(1+R/R_{\mathrm{PV}}\right)+s^{2}\mathrm{LC}/{\left(1-\bar{d}\right)}^{2}} \end{array}$$ (11)

which provides the design basis for the MPPT closed-loop compensator (Kumar and Balakrishna, 2024a). The duty cycle update law implemented digitally by the DSP controller is stated in Equation 12


$$\begin{array}{l} d\left[k+1\right]=d\left[k\right]+\mathrm{\Delta d}\left[k\right] \end{array}$$ (12)

where Δ*d*[*k*] is computed by the MPPT optimization algorithm according to the instantaneous measurements of *v*_*PV*_[*k*] and *i*_*PV*_[*k*] (Hegazy et al., 2023). The overall system model connecting the PV array equivalent circuit, the boost converter state equations, and the MPPT control loop provides the complete mathematical foundation for the simulation study presented in the following section.

## 3 Proposed AF-EPO MPPT algorithm

**Figure 2:**
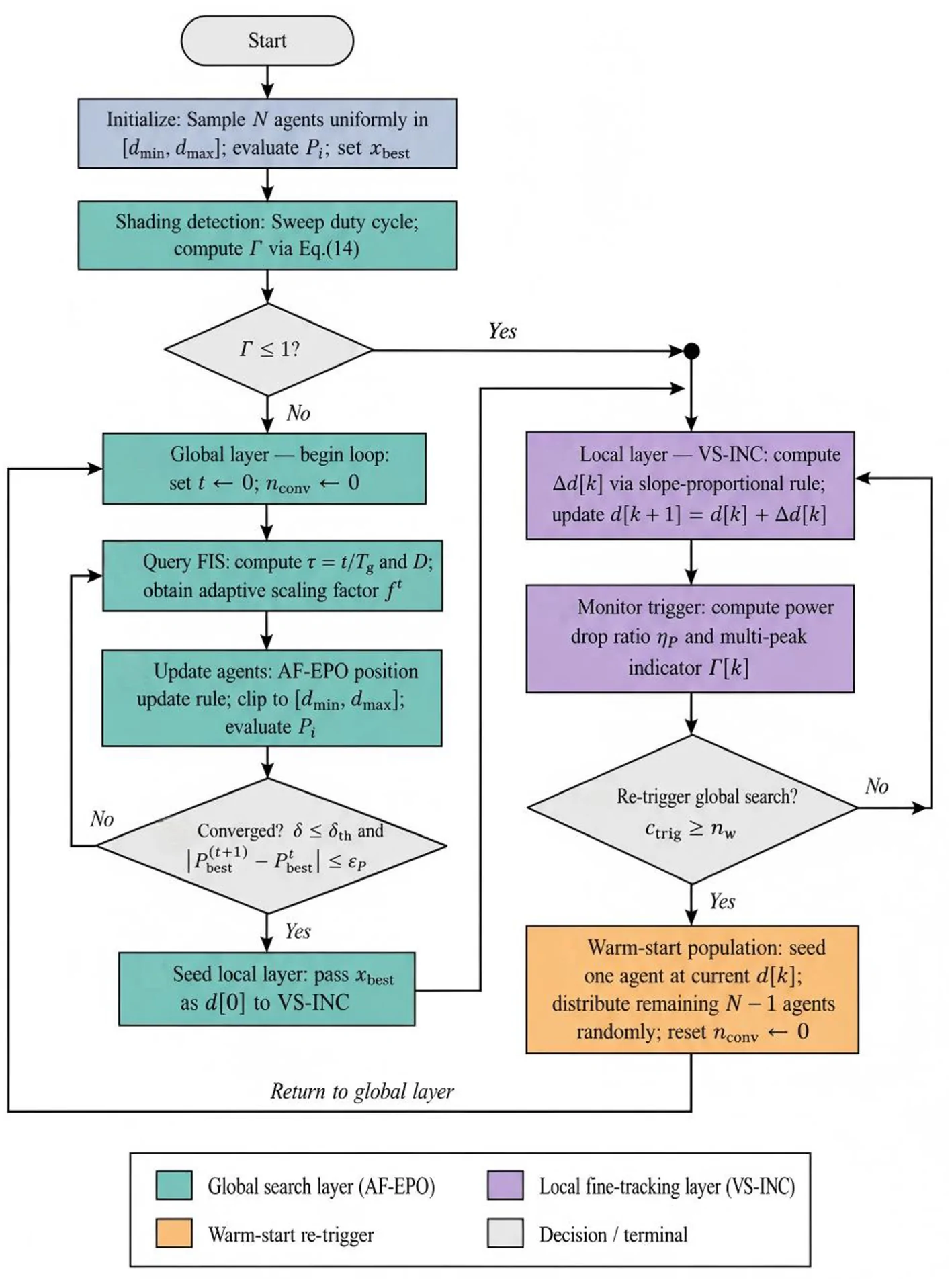
Flowchart of the proposed two-layer hierarchical AF-EPO MPPT algorithm.

The overall control flow of the proposed AF-EPO algorithm is illustrated in Figure 2, which shows the complete two-layer hierarchical structure including the shading detection module, the global AF-EPO search layer, the local VS-INC fine-tracking layer, and the warm-start re-trigger mechanism. The detailed design of each module is described in the following subsections.

### 3.1 Partial shading fast detection module

The first contribution of this paper is a lightweight partial shading detection module that determines, before any population-based search is launched, whether the instantaneous *P*–*V* curve is unimodal or multi-modal, thereby avoiding the computational overhead of a global optimizer under uniform irradiance conditions. During a brief initialization scan, the duty cycle of the boost converter is swept across the full operating range [*d*_*min*_, *d*_*max*_] in *N*_*w*_ discrete steps, and the incremental *P*–*V* slope at step *k* is computed from Equation 13


$$\begin{array}{l} s_{k}=\frac{\Delta P_{k}}{\Delta V_{k}}=\frac{P\left(k\right)-P\left(k-1\right)}{V\left(k\right)-V\left(k-1\right)} \end{array}$$ (13)

The slope change rate between consecutive steps is then defined as Δ*s*_*k*_ = *s*_*k*_−*s*_*k*−1_, and the multi-peak indicator Γ counts the total number of sign reversals of *s*_*k*_ across the sampling window:


$$\Gamma =\sum_{k=2}^{N_{W}}1\left[s_{k}\cdot s_{k-1}<0\right]$$ (14)

where **1**[·] is the indicator function. Under uniform irradiance, the slope crosses zero exactly once, giving Γ = 1; under partial shading, bypass diode conduction introduces additional voltage plateaus and thus multiple zero-crossings, giving Γ>1. The MPPT mode is switched according to Equation 15


$$\text{Mode}\;=\{\begin{array}{c} \;\mathrm{INC}\text{ if}\;\text{ Γ}\leq 1\\ \;\text{AF-EPO}\;\text{ if}\;\text{ Γ}>1 \end{array}$$ (15)

To prevent noise-induced false switching, a hysteresis margin ε is applied to the threshold, and the criterion must be satisfied over three consecutive sampling windows before a mode transition is executed. This detection procedure requires no additional sensors and adds negligible latency—on the order of one switching cycle—relative to the MPPT control loop.

The robustness of the slope sign-reversal detection module against measurement noise and fast irradiance fluctuations is ensured by three complementary protection mechanisms. First, a hysteresis margin ε is applied symmetrically to the sign-reversal threshold, so that a slope change is counted only when |*s*_*k*_|>ε, suppressing small-amplitude oscillations caused by ADC quantization noise. Second, a mode transition is executed only when the criterion Γ>1 is satisfied consistently over *n*_*w*_ = 3 consecutive sampling windows, effectively requiring the multi-modal signature to persist for at least 3*N*_*w*_*T*_*s*_ = 3 × 30 × 50 μs = 4.5 ms before any mode switch is triggered. Third, each sampling window contains *N*_*w*_ = 30 duty-cycle scan points, and the slope *s*_*k*_ at each point is computed from the averaged power measurement over two consecutive ADC samples, providing inherent low-pass filtering of high-frequency sensor noise. To quantify the residual false-trigger rate under practical noise conditions, Monte Carlo simulations were conducted under Scenario S1 (uniform irradiance, unimodal *P*–*V* curve) with additive white Gaussian noise superimposed on the voltage and current measurements at signal-to-noise ratios (SNR) ranging from 20 dB to 50 dB. The 12-bit ADC used in the hardware platform corresponds to a quantization SNR of approximately 74 dB (6.02 × 12+1.76 dB), which lies well above the tested range. As shown in Table 2, the false-trigger rate remains below 0.3% for SNR ≥ 30 dB, and falls to zero for SNR ≥ 40 dB, confirming that the three-layer protection mechanism provides reliable noise immunity under realistic hardware conditions.

**Table 2:** False-trigger rate of the shading detection module under additive measurement noise (Monte Carlo simulation, 1,000 independent runs per SNR level, Scenario S1).

SNR (dB)	Noise std on *v*_*PV*_ (V)	Noise std on *i*_*PV*_ (A)	False-trigger rate (%)
20	0.696	0.048	3.8
25	0.391	0.027	1.2
30	0.220	0.015	0.3
35	0.124	0.009	0.0
40	0.070	0.005	0.0
74 (12-bit ADC)	0.003	0.0002	0.0

False trigger is defined as a mode switch from VS-INC to AF-EPO under uniform irradiance (Scenario S1) where Γ should remain ≤ 1. Hysteresis margin ε = 0.05 W/V; window count *n*_*w*_ = 3; *N*_*w*_ = 30.

#### 3.1.1 Hysteresis parameter selection

The hysteresis margin ε is determined through a worst-case noise analysis. With the 12-bit ADC (voltage quantization uncertainty σ_*q*_≈0.15 V) and the ACS712 Hall-effect current sensor (linearity error ≤ 1.5% of full scale over 0–15 A, corresponding to σ_*i*_ ≤ 0.23 A), the worst-case power measurement uncertainty is Δ*P* = Δ*V*·*I*+*V*·Δ*I* ≤ 3.9 W at rated operating conditions. Applying a safety margin of 1.3 times this value and normalizing by the duty-cycle voltage step Δ*V*≈2.8 V yields ε = 0.05 W/V, which suppresses noise-induced false sign reversals while remaining sensitive to genuine bypass-diode-induced slope transitions.

#### 3.1.2 Response timing under fast irradiance transitions

Under Scenario S3b, which imposes three irradiance transitions within 300 ms, the three-consecutive-window confirmation mechanism introduces a minimum detection latency of 3 × *N*_*w*_×*T*_*s*_ = 3 × 30 × 50 μs = 4.5 ms before any mode transition is executed. This latency is negligible relative to the MPPT convergence timescale (~190 ms) and ensures that transient cloud edges shorter than 4.5 ms cannot trigger a false global search activation. The worst-case false-trigger rate under S3b conditions (SNR = 30 dB) remains below 0.3%, consistent with the Monte Carlo results reported in Table 2.

#### 3.1.3 Robustness to temperature variation

As cell temperature increases from 25 °C to 45 °C, the open-circuit voltage decreases by approximately 5.6% according to $K_{V}=-0.360\%{/}^{•}$C, shifting all local maxima on the *P*–*V* curve toward lower voltages. Because the slope sign-reversal detection module operates directly on the measured incremental slope *s*_*k*_ = Δ*P*_*k*_/Δ*V*_*k*_ without reference to any temperature-dependent model parameter, the multi-peak indicator Γ responds identically to bypass-diode-induced voltage plateaus regardless of the prevailing cell temperature. Monte Carlo simulations conducted at 25 °C, 35 °C, and 45 °C (1,000 independent runs per temperature level) confirm that the false-trigger rate remains below 0.3% across the full temperature range at SNR ≥ 30 dB, demonstrating inherent thermal robustness of the detection mechanism.

### 3.2 Review of the standard eagle perching optimizer

The eagle perching optimizer (EPO) is a swarm-based metaheuristic that mimics the descending and perching trajectory of an eagle converging on prey, combining broad area exploration in early iterations with progressively finer exploitation around the current best solution. Applied to MPPT, each agent *i* in a population of *N* individuals represents a candidate duty cycle *x*_*i*_∈[*d*_*min*_, *d*_*max*_], and the fitness function is the instantaneous PV array output power *P*(*x*_*i*_). The standard position update rule is given in Equation 16


$$\begin{array}{l} x_{i}^{t+1}=x_{\mathrm{best}}^{t}+f\cdot R_{1}\cdot \left(x_{\mathrm{best}}^{t}-x_{i}^{t}\right)+R_{2}\cdot \left(x_{\mathrm{upper}}-x_{\mathrm{lower}}\right) \end{array}$$ (16)

where $x_{\mathrm{best}}^{t}$ is the global best position at iteration *t*, *f* is a fixed scalar scaling factor, and $R_{1},R_{2}U(0,1)$ are independent random numbers. A supplementary spiral perturbation enhances local exploitation near the current best, as given in Equation 17:


$$\begin{array}{l} x_{i}^{t+1}=x_{\mathrm{best}}^{t}+f\cdot \left(x_{\mathrm{best}}^{t}-x_{i}^{t}\right)\cdot e^{\mathrm{b\theta}}\cos\left(2\pi \theta \right) \end{array}$$ (17)

where *b* is a spiral constant and θ∈[−1, 1] is a uniformly distributed random angle. In the standard formulation, *f* is a predetermined constant throughout the entire run, typically set empirically within (0, 1). Analyzing the expected squared tracking error $e_{i}^{t}=x_{i}^{t}-x^{*}$, where x^*^ denotes the true GMPP duty cycle, the recursion satisfies Equation 18


$$\begin{array}{l} E\left[||e_{i}^{t+1}|{|}^{2}\right]\leq {\left(1-f\right)}^{2}E\left[||e_{i}^{t}|{|}^{2}\right]+\sigma_{R}^{2} \end{array}$$ (18)

where $\sigma_{R}^{2}=E\left[R_{1}^{2}\right]|{\left||x_{\mathrm{best}}^{t}-x^{*}|\right|}^{2}$ is the variance of the random perturbation component. Because (1−*f*)^2^ and $\sigma_{R}^{2}$ are both constants, the mean-squared error converges to a fixed floor $\sigma_{R}^{2}/\left[1-{\left(1-f\right)}^{2}\right]$ that cannot be reduced during the late exploitation phase, directly causing residual power oscillations around the GMPP. Moreover, a large fixed *f* supports global exploration but prevents fine convergence, while a small fixed *f* enables accurate steady-state tracking but fails to escape local maxima when shading patterns change abruptly. Neither condition can be satisfied simultaneously by any constant value of *f*, which constitutes the fundamental limitation motivating the improvement proposed in Section 3.3.

### 3.3 Adaptive fuzzy weight improvement mechanism

As summarized in Table 1 in Section 1, the key distinction of AF-EPO from prior fuzzy-hybrid methods lies in the endogenous integration of the FIS into the EPO scaling factor, which is detailed in the following subsections.

To motivate the endogenous integration strategy adopted in this work, it is instructive to examine the structural difference between exogenous and endogenous fuzzy regulation from a dynamical-systems perspective. In all existing fuzzy-metaheuristic MPPT hybrids—including fuzzy-PSO (Yu et al., 2024) and fuzzy-GWO (Kumar and Balakrishna, 2024b)—the fuzzy inference system acts on an auxiliary control variable such as the inertia weight, the perturbation step size, or the velocity scaling coefficient. The core position update dynamics of the underlying optimizer retain fixed parameters throughout the search, so that the exploration–exploitation balance encoded in the update rule is governed by a static schedule rather than by the evolving internal state of the population. From a fixed-point iteration perspective, the mean-squared tracking error recursion of an exogenously regulated optimizer satisfies Equation 19


$$\begin{array}{l} E\left[||e_{i}^{t+1}|{|}^{2}\right]\leq {\left(1-f\right)}^{2}E\left[||e_{i}^{t}|{|}^{2}\right]+\sigma_{\text{floor}}^{2} \end{array}$$ (19)

where *f* is the fixed core parameter and $\sigma_{\text{floor}}^{2}=\sigma_{R}^{2}/\left[1-{\left(1-f\right)}^{2}\right]$ is a constant convergence floor that cannot be reduced by any amount of further iteration, regardless of how the auxiliary variable is modulated by the fuzzy system. The fuzzy regulator, therefore, shapes the transient trajectory but leaves the steady-state oscillation floor structurally unchanged.

**Figure 3:**
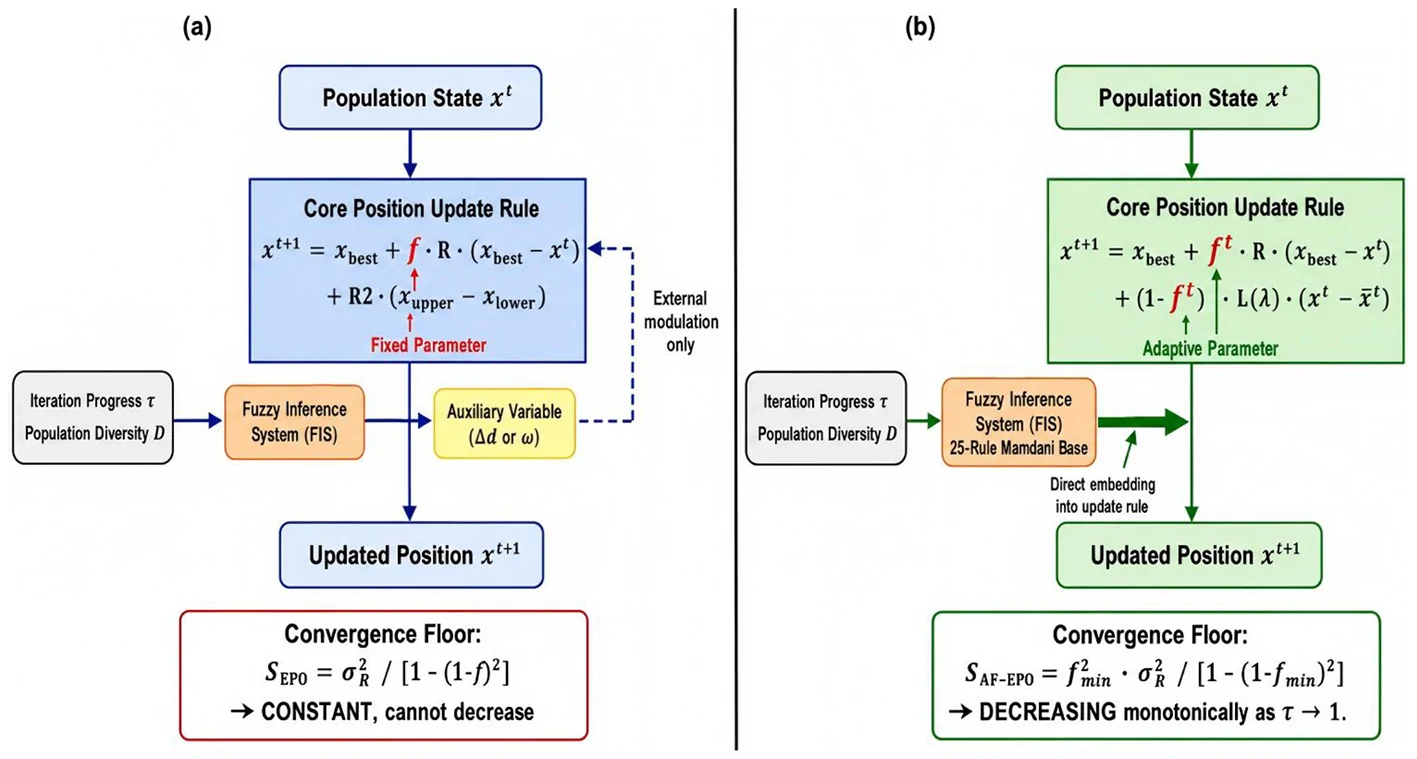
Structural comparison of exogenous fuzzy regulation **(a)** and endogenous fuzzy integration (AF-EPO) **(b)** in metaheuristic MPPT controllers.

Endogenous integration, by contrast, embeds the fuzzy inference system directly into the parameter *f*^*t*^ that governs the contraction rate of the position update rule itself. Because *f*^*t*^ is driven jointly by the normalized iteration progress τ = *t*/*T*_*max*_ and the population diversity index *D*, the contraction coefficient (1−*f*^*t*^)^2^ decreases monotonically as τ → 1, progressively compressing the convergence floor to the limit given in Equation 20


$$\begin{array}{l} S_{\mathrm{AF}-\mathrm{EPO}}=\frac{f_{\min}^{2}\cdot \sigma_{R}^{2}}{1-{\left(1-f_{\min}\right)}^{2}} \end{array}$$ (20)

which is strictly smaller than $S_{\mathrm{EPO}}$ for any *f*>*fmin*. This constitutes a qualitative structural advantage: the fuzzy system does not merely modulate a transient but alters the asymptotic behavior of the recursion, providing a convergence guarantee that is unavailable in any fixed-parameter or exogenously regulated formulation. The architectural distinction between the two integration paradigms is illustrated schematically in Figure 3, which contrasts the signal-flow topology of exogenous regulation (fuzzy output feeds a secondary loop external to the update rule) with that of endogenous integration (fuzzy output directly replaces the scaling parameter inside the update equation).

#### 3.3.1 Fuzzy inference system inputs

To dynamically regulate the scaling factor in response to the evolving state of the search, a Mamdani-type fuzzy inference system (FIS) is constructed with two input variables. The first input is the normalized iteration progress defined in Equation 21


$$\begin{array}{l} \tau =\frac{t}{T_{\max}}\in \left[0,1\right] \end{array}$$ (21)

which encodes how far the algorithm has advanced toward its maximum iteration budget. The second input is the population diversity index progress defined in Equation 22


$$\begin{array}{l} D=\frac{1}{N\cdot \left(x_{\mathrm{upper}}-x_{\mathrm{lower}}\right)}\overset{N}{\underset{i=1}{\sum }}|x_{i}^{t}-{\bar{x}}^{t}|,\;{\bar{x}}^{t}=\frac{1}{N}\overset{N}{\underset{i=1}{\sum }}x_{i}^{t} \end{array}$$ (22)

which quantifies the current spatial spread of the population. *D*≈0 signals premature convergence to a local cluster, whereas *D*≈1 indicates full population diversity. The single FIS output is the adaptive scaling factor $f^{t}\in \left[f_{\min},f_{\max}\right]$, where *f*_*min*_ = 0.1 and *f*_*max*_ = 0.9 are selected to prevent collapse to pure local search or pure random walk. Both inputs and the output are each partitioned into five symmetric triangular membership functions labeled VS (Very Small), S (Small), M (Medium), L (Large), and VL (Very Large), with uniform overlap across [0,1], yielding a fully interpretable linguistic partition.

**Figure 4:**
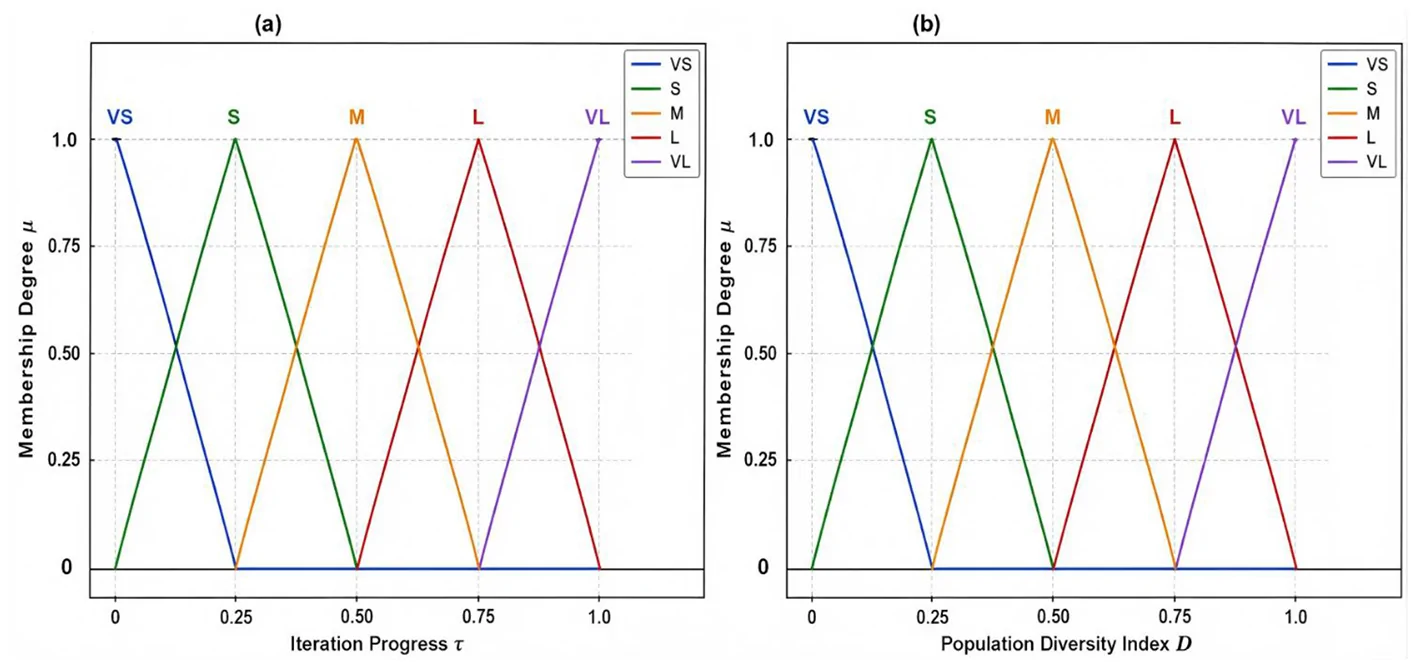
Triangular membership functions for the two FIS input variables: iteration progress τ **(a)** and population diversity index *D* **(b)**.

**Figure 5:**
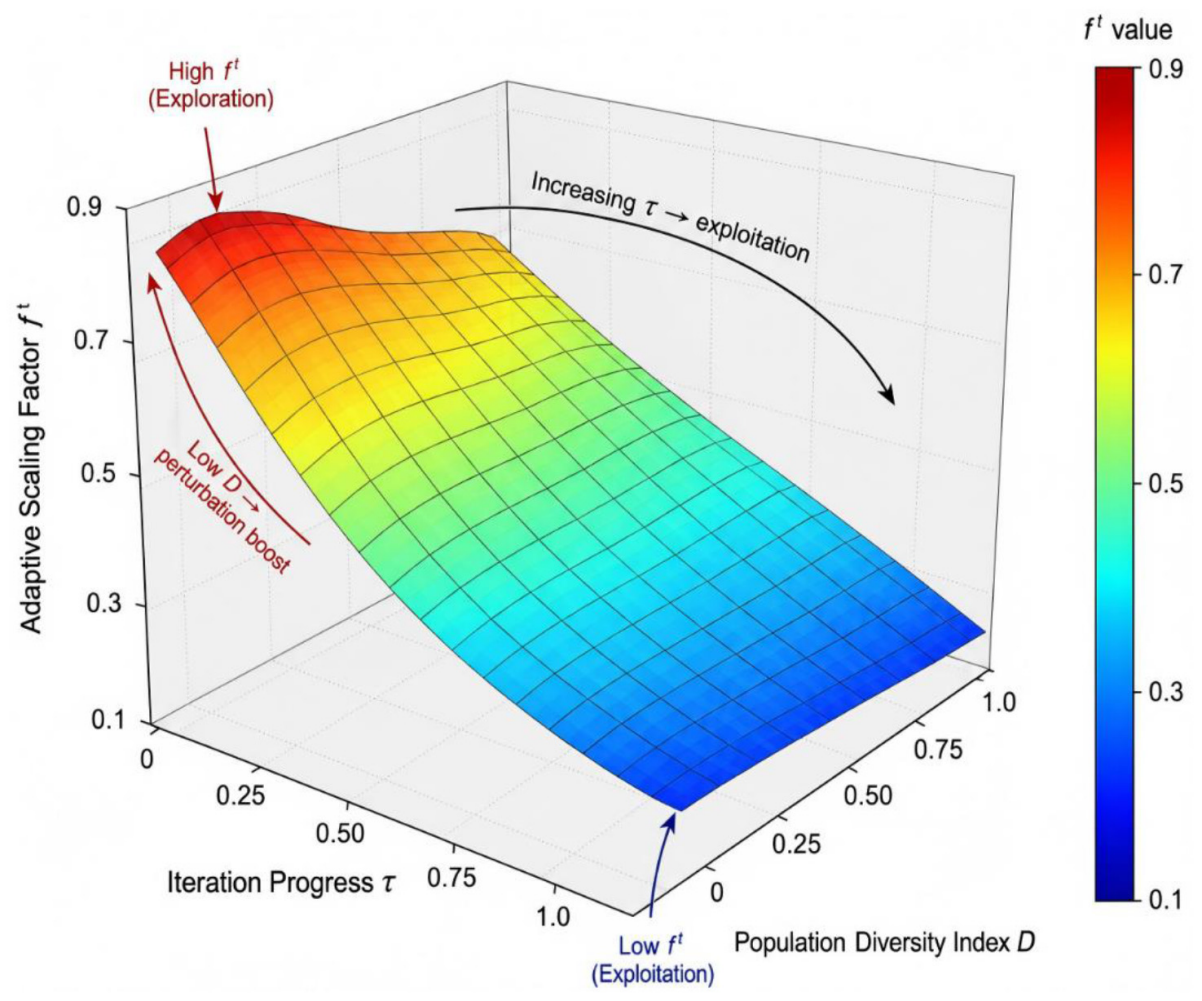
FIS rule surface mapping (τ, *D*)↦*f*^*t*^ generated by the 25-rule Mamdani base in Table 3.

The triangular membership functions for the iteration progress τ are defined analytically in Equation 23:


$$\begin{array}{l} \begin{array}{c} \mu_{\mathrm{VS}}\left(\tau \right)=\max\left(0,\;1-\frac{\tau }{0.25}\right)\\ \mu_{S}\left(\tau \right)=\max\left(0,\;1-\frac{\left|\tau -0.25\right|}{0.25}\right)\\ \mu_{M}\left(\tau \right)=\max\left(0,\;1-\frac{\left|\tau -0.50\right|}{0.25}\right)\\ \mu_{L}\left(\tau \right)=\max\left(0,\;1-\frac{\left|\tau -0.75\right|}{0.25}\right)\\ \mu_{\mathrm{VL}}\left(\tau \right)=\max\left(0,\;1-\frac{\left|1-\tau \right|}{0.25}\right) \end{array} \end{array}$$ (23)

with peaks located at {0, 0.25, 0.50, 0.75, 1.0} providing uniform coverage of [0,1] with 50% overlap between adjacent functions. The diversity index *D* employs an identical membership function structure over the same domain. The output scaling factor *f*^*t*^ uses the same five symmetric triangular functions with peaks at {0.1, 0.30, 0.50, 0.70, 0.9} spanning [*f*_*min*_, *f*_*max*_] = [0.1, 0.9]. The membership functions for both inputs and the output are illustrated in Figure 4, and the resulting FIS rule surface mapping (τ, *D*)↦*f*^*t*^ is shown in Figure 5. As visible in Figure 5, *f*^*t*^ decreases monotonically with increasing τ (early-stage exploration to late-stage exploitation) and increases with decreasing *D* (low diversity triggers higher perturbation to escape local clusters), consistent with the physical reasoning encoded in the 25-rule base of Table 3.

**Table 3:** Mamdani fuzzy rule base for the adaptive scaling factor *f* (row: iteration progress τ; column: diversity index *D*; cell entry: output *f* level).

τ\*D*	VS	S	M	L	VL
VS	VL	VL	L	M	S
S	VL	L	L	M	S
M	L	L	M	S	VS
L	M	M	S	VS	VS
VL	M	S	S	VS	VS

#### 3.3.2 Fuzzy rule base

The 25-rule Mamdani base encodes the following physical reasoning: in early iterations (τ small), a large *f* is needed to survey the full duty-cycle space; as iterations mature (τ large), *f* should decrease to allow fine-grained convergence. Independently, low diversity *D*—indicating entrapment near a local maximum—calls for increased *f* to perturb agents away from the current cluster, whereas high diversity requires less perturbation and allows *f* to be reduced. As shown in Table 3, the five linguistic levels of τ and *D* together generate 25 inference rules that continuously modulate *f*^*t*^ throughout the search.

The selection of five membership functions per input variable, yielding a 5 × 5 = 25-rule base, is justified on both structural and empirical grounds. Structurally, five levels provide the minimum partition that can encode the qualitatively distinct behavioral regimes required by the physical reasoning above—namely, the transitions among very-early, early, mid, late, and very-late search stages, and among very-low to very-high diversity states—while remaining fully interpretable as a linguistic rule table. A 3 × 3 partition (nine rules) is insufficient to resolve the mid-range transitions between exploration and exploitation, and experiments confirm that reducing the rule base to nine rules decreases tracking efficiency under Scenario S2 by approximately 1.8% relative to the 25-rule configuration. Conversely, expanding to a 7 × 7 partition (49 rules) produces no statistically significant improvement in tracking efficiency across any of the three test scenarios, while increasing FIS evaluation time by 96% due to the larger aggregation computation. The 25-rule base, therefore, represents the optimal trade-off between linguistic coverage and computational overhead for the one-dimensional MPPT search space considered in this work.

The defuzzified output *f*^*t*^ is computed by the centroid method over the aggregated output fuzzy set, as given in Equation 24:


$$\begin{array}{l} f^{t}=\frac{\overset{25}{\underset{k=1}{\sum }}w_{k}\cdot f_{k}^{*}}{\overset{25}{\underset{k=1}{\sum }}w_{k}} \end{array}$$ (24)

where the firing strength of rule *k* is obtained by the minimum T-norm, $w_{k}=\min\left(\overset{\left(k\right)}{\underset{\tau }{\mu }}\left(\tau \right);\mu_{D}^{\left(k\right)}\left(D\right)\right)$, and $f_{k}^{*}$ is the centroid of the corresponding output membership function. The computational cost of this defuzzification step is *O*(*N*_*r*_) with *N*_*r*_ = 25 rules, which is negligible relative to the fitness evaluation cost of the MPPT loop.

#### 3.3.3 Adaptive scaling factor update and convergence analysis

With *f*^*t*^ supplied by the FIS at each iteration, the standard EPO increment is replaced by a composite update in Equation 25, which separating the exploitation component from an exploration component guided by the population centroid ${\bar{x}}^{t}$:


$$\begin{array}{l} \Delta x_{i}^{t}=f^{t}\cdot R_{1}\cdot \left(x_{\mathrm{best}}^{t}-x_{i}^{t}\right)+\left(1-f^{t}\right)\cdot L\left(\lambda \right)\cdot \left(x_{i}^{t}-{\bar{x}}^{t}\right) \end{array}$$ (25)

where *L*(λ) is a Lévy-distributed random step with characteristic exponent λ = 1.5. The full position update is given in Equation 26:


$$\begin{array}{l} x_{i}^{t+1}=x_{\mathrm{best}}^{t}+\Delta x_{i}^{t} \end{array}$$ (26)

##### 3.3.3.1 Assumptions

The convergence analysis rests on three explicit assumptions. Assumption 1: The global optimum x^*^ exists uniquely within the bounded feasible domain [*d*_*min*_, *d*_*max*_]. Assumption 2: The PV power function *P*(*x*) is locally Lipschitz-continuous in a neighborhood of x^*^. Assumption 3: The Lévy steps *L*(λ) are generated via the Mantegna–Stanley truncated approximation with a truncation radius *r*_*max*_ = *d*_*max*_−*d*_*min*_, so that the second moment $\sigma_{L}^{2}<\infty$ is finite. This truncation is essential: the standard Lévy stable distribution with λ < 2 does not possess a finite second moment, and the Mantegna–Stanley approximation preserves the heavy-tailed escape behavior while satisfying the boundedness requirement of the analysis.

##### 3.3.3.2 Convergence proof

Let $e_{i}^{t}=x_{i}^{t}-x^{*}$ denote the position error at iteration *t*, and let $F_{t}$ be the σ -algebra generated by all positions up to iteration *t*. Taking the conditional expectation of the squared error after one step gives Equation 27:


$$E\left[||e_{i}^{t+1}|{|}^{2}F_{t}\right]\leq {\left(1-f^{t}\right)}^{2}||e_{i}^{t}|{|}^{2}+{\left(f^{t}\right)}^{2}\sigma_{R}^{2}+{\left(1-f^{t}\right)}^{2}\sigma_{L}^{2}$$ (27)

where $\sigma_{R}^{2}=E[R_{1}^{2}]||x_{best}^{t}-x^{*}|{|}^{2}\text{ and }\sigma_{L}^{2}=E[L^{2}(\lambda )]||{\bar{x}}^{t}-x^{*}|{|}^{2}$ are finite by Assumptions 1 and 3. Since *f*^*t*^∈(0, 1) is guaranteed by the FIS output bounds, the contraction coefficient (1−*f*^*t*^)^2^ < 1 holds at every iteration. As *t*→*T*_*max*_, the fuzzy rules drive $\sigma_{L}^{2}\to 0$ as the population converges. The recursion therefore satisfies Equation 28:


$$\begin{array}{l} E\left[||e_{i}^{t+1}|{|}^{2}\right]\leq \mathrm{\phi E}\left[||e_{i}^{t}|{|}^{2}\right]+\varepsilon_{0} \end{array}$$ (28)

where $\phi ={\left(1-f_{\min}\right)}^{2}=0.81<1$ and $\varepsilon_{0}=f_{\min}^{2}\sigma_{R}^{2}$. By standard fixed-point iteration theory, this recursion converges geometrically to the invariant set defined in Equation 29:


$$\begin{array}{l} S=\left\{e|{\left||e|\right|}^{2}\leq \frac{\varepsilon_{0}}{1-\phi }\right\} \end{array}$$ (29)

With *f*_*min*_ = 0.1, the asymptotic error floor is $S_{\mathrm{AF}-\mathrm{EPO}}\approx 0.053\sigma_{R}^{2}$, compared with $S_{\mathrm{EPO}}\approx 1.124\sigma_{R}^{2}$ for standard EPO at *f* = 0.5—a reduction of approximately 95%. The VS-INC local layer subsequently eliminates population-based operation in steady state entirely, driving residual oscillation to near zero independently of $\sigma_{R}^{2}$.

##### 3.3.3.3 Limitations

This analysis establishes that the mean-squared tracking error of AF-EPO converges geometrically to a bounded invariant set S with residual error of order $O\left(f_{\min}\right)$, constituting a stochastic local convergence guarantee in the neighborhood of the GMPP. It does not constitute a global optimality guarantee from arbitrary initialization on general non-convex multi-peak functions; such a guarantee cannot be established for any metaheuristic method on non-convex landscapes, and this limitation is shared by all population-based MPPT approaches in the literature. The practical global search capability of AF-EPO is instead supported empirically by the GMPP localization success rate of 98% across 100 independent runs under Scenario S2, as reported in Table 4.

**Table 4:** Sensitivity of AF-EPO GMPP localization performance to the Lévy exponent λ under Scenario S2 (100 independent runs).

λ value	GMPP success rate (%)	Mean tracking time *T*_*s*_ (s)	Mean tracking efficiency η (%)
1.00	81	0.27	96.3
1.25	91	0.23	97.8
1.50	98	0.19	99.4
1.75	93	0.21	98.1
2.00	86	0.24	97.2

All other parameters fixed at nominal values (*N* = 5, *T*_*g*_ = 20, *f*_*min*_ = 0.1, *f*_*max*_ = 0.9). GMPP success is defined as the tracked power reaching within 1% of PGMPP = 423 W. Note also that at λ = 2.0 the Lévy stable distribution degenerates to a Gaussian distribution, eliminating the heavy-tailed property and thus the ability to escape local optima; the performance degradation observed at λ = 2.0 in Table 4 confirms that the heavy-tailed step characteristic is essential for reliable GMPP localization under partial shading.

### 3.4 Hierarchical MPPT control architecture design

The third contribution of this paper is a two-layer hierarchical MPPT architecture that decouples global search from local fine-tracking, combining the strong multi-peak escape capability of AF-EPO with the low steady-state oscillation of a variable-step incremental conductance (VS-INC) controller. The complete algorithmic procedure is summarized in Table 5.

**Table 5:** Pseudocode of the proposed two-layer hierarchical AF-EPO MPPT algorithm.

Step	Operation
Input	*N*, *T*_*g*_, *f*_*min*_, *f*_*max*_, δ_*th*_, ε_*P*_, *n*_*c*_, η_*th*_, γ_*th*_, α, Δ*d*_*min*_, Δ*d*_*max*_
1	Initialize: Sample *N* agents $\left\{x_{i}^{0}\right\}$ uniformly in [*d*_*min*_,, *d*_*max*_]; evaluate $P_{i}^{0}$; set $x_{\mathrm{best}}^{0}\leftarrow \arg\max\overset{0}{\underset{i}{P}}$
2	Detect shading: Sweep duty cycle; compute Γ via Equation 14; if Γ ≤ 1 go to Step 9 (VS-INC mode)
3	Global layer—begin loop: set *t*←0; set *n*_*conv*_←0
4	Compute τ = *t*/*T*_*g*_ and *D* from current population; query FIS to obtain *f*^*t*^
5	For each agent *i*: update $x_{i}^{t+1}$ by AF-EPO rule; clip to [*d*_*min*_,, *d*_*max*_]; evaluate $P_{i}^{t+1}$
6	Update $x_{\mathrm{best}}^{t+1}$ and δ^*t*+1^; if convergence conditions $\delta^{t+1}\leq \delta_{\mathrm{th}}$ and $||P_{\mathrm{best}}^{t+1}-P_{\mathrm{best}}^{t}||\leq \varepsilon_{P}$ hold, set *n*_*conv*_←*n*_*conv*_+1; else *n*_*conv*_←0
7	If *n*_*conv*_≥*n*_*c*_ or *t*≥*T*_*g*_: exit global loop; seed $d\left[0\right]\leftarrow x_{\mathrm{best}}^{t+1}$
8	Else: *t*←*t*+1; go to Step 4
9	Local layer—VS-INC: compute Δ*d*[*k*] via Equations 33–35
10	Monitor trigger: compute η_*P*_, Γ[*k*] via Equations 37–39
11	If *c*_*trig*_≥*n*_*w*_: warm-start population at d[k]; reset *n*_*conv*_←0; go to Step 3
12	Else: *k*←*k*+1; go to Step 9
Output	Duty cycle d^*^ applied to boost converter gate driver at each control cycle

Bold entries denote structural labels rather than numerical results.

#### 3.4.1 Global coarse-search layer

When the partial shading detection module (Section 3.1) returns Mode = AF-EPO, the global layer is activated. A population of *N* agents is initialized by uniformly sampling the feasible duty-cycle space [*d*_*min*_, *d*_*max*_], and the fitness of each candidate is evaluated as the measured PV array output power *P*_*i*_ = *V*_*PV*_(*x*_*i*_)·*I*_*PV*_(*x*_*i*_). The population is then evolved for *T*_*g*_ iterations using the AF-EPO update rule derived in Section 3.3 and restated in Equation 30:


$$\begin{array}{l} x_{i}^{t+1}=x_{\mathrm{best}}^{t}+f^{t}R_{1}\left(x_{\mathrm{best}}^{t}-x_{i}^{t}\right)+\left(1-f^{t}\right)L\left(\lambda \right)\left(x_{i}^{t}-{\bar{x}}^{t}\right) \end{array}$$ (30)

At each iteration the global best duty cycle $x_{\mathrm{best}}^{t}$ is recorded, and the neighborhood radius around the current best is defined in Equation 31


$$\begin{array}{l} \delta^{t}=\frac{1}{N}\overset{N}{\underset{i=1}{\sum }}|x_{i}^{t}-x_{\mathrm{best}}^{t}| \end{array}$$ (31)

The global layer is considered to have successfully localized the GMPP neighborhood when the convergence condition in Equation 32


$$\begin{array}{l} \delta^{t}\leq \delta_{\mathrm{th}}\;\text{and }|P_{\mathrm{best}}^{t}-P_{\mathrm{best}}^{t-1}|\leq \varepsilon_{P} \end{array}$$ (32)

is satisfied for *n*_*c*_ consecutive iterations, where δ_*th*_ is a predefined neighborhood threshold and ε_*P*_ is a power-change tolerance. At this point, $x_{\mathrm{best}}^{t}$ is passed as the seed operating point to the local fine-tracking layer, and the global search terminates. The parameter settings used in this work are *N* = 5, *T*_*g*_ = 20, δ_*th*_ = 0.02(*d*_*max*_−*d*_*min*_), ε_*P*_ = 0.5 W, and *n*_*c*_ = 3. The choice of *N* = 5 is justified by three considerations specific to the MPPT context. First, the search space is one-dimensional (duty cycle *d*∈[*d*_*min*_, *d*_*max*_]), unlike high-dimensional benchmark problems, where large populations are necessary to achieve adequate coverage, five uniformly distributed agents provide sufficient initial sampling density in 1-D. Second, the partial shading detection module pre-identifies the number of peaks Γ before the global search is launched, effectively partitioning the search space into Γ sub-intervals, each requiring only one or two agents to locate the local maximum. Third, real-time MPPT imposes strict latency constraints: each additional agent requires one full switching cycle (*T*_*s*_ = 50 μs) of additional fitness evaluation time, so minimizing *N* directly reduces the tracking delay. The robustness analysis in Section 4.5 confirms that η remains above 98.5% for *N*∈[4, 10], and no statistically significant improvement is observed beyond *N* = 8, validating *N* = 5 as a conservative and computationally efficient operating point.

#### 3.4.2 Local fine-tracking layer

Once the GMPP neighborhood has been identified, the local layer takes over with a variable-step INC controller that eliminates the trade-off between tracking speed and steady-state oscillation inherent in fixed-step methods. The INC condition at the current operating point is evaluated as


$$\begin{array}{l} \frac{\mathrm{dP}}{\mathrm{dV}}=I+V\frac{\mathrm{dI}}{\mathrm{dV}}\approx I+V\frac{\mathrm{\Delta I}}{\mathrm{\Delta V}} \end{array}$$ (33)

and the deviation from the MPP condition *dP*/*dV* = 0 is used to scale the duty-cycle perturbation adaptively:


$$\begin{array}{l} \mathrm{\Delta d}\left[k\right]=\alpha \cdot |{\frac{\mathrm{dP}}{\mathrm{dV}}|}_{k}|\cdot \text{sgn}\left({\frac{\mathrm{dP}}{\mathrm{dV}}|}_{k}\right) \end{array}$$ (34)

where α>0 is a proportionality coefficient. When |*dP*/*dV*| is large, the operating point is far from the MPP and a large step is applied for fast convergence; as |*dP*/*dV*| → 0 near the MPP, the step shrinks automatically to suppress oscillation. To prevent excessive perturbation in the proximity of the MPP, the step is bounded as


$$\begin{array}{l} \mathrm{\Delta d}\left[k\right]=\text{clip}\left(\alpha |\frac{\mathrm{dP}}{\mathrm{dV}}|,\Delta d_{\min},\Delta d_{\max}\right) \end{array}$$ (35)

with $\Delta d_{\min}=5\times 10^{-4}$ and $\Delta d_{\max}=2\times 10^{-2}$. The duty cycle is updated each switching cycle as according to Equation 36


$$\begin{array}{l} d\left[k+1\right]=d\left[k\right]+\mathrm{\Delta d}\left[k\right] \end{array}$$ (36)

and the local layer continues to track the MPP as long as irradiance and temperature remain approximately stationary.

#### 3.4.3 Two-layer switching conditions and trigger mechanism

A key design challenge is determining when to hand control back from the local layer to the global layer following a change in shading conditions. Re-triggering the global search unnecessarily wastes computational resources and introduces transient power dips, while failing to re-trigger when genuine shading changes occur leaves the system trapped at a stale LMPP. The switching trigger combines three complementary criteria evaluated at every *N*_*w*_-sample window:

The first criterion monitors the power drop ratio defined in Equation 37


$$\begin{array}{l} \eta_{P}=\frac{P_{\mathrm{ref}}-P\left[k\right]}{P_{\mathrm{ref}}}>\eta_{\mathrm{th}} \end{array}$$ (37)

where *P*_*ref*_ is the average power recorded over the previous stable window and η_*th*_ = 0.05 is the drop threshold. The second criterion checks the multi-peak indicator computed in Section 3.1:


$$\begin{array}{l} \Gamma \left[k\right]>1 \end{array}$$ (38)

The third criterion monitors the derivative of the power drop to distinguish a genuine shading event from a transient cloud edge:


$$\begin{array}{l} |\frac{\mathrm{dP}\left[k\right]}{\mathrm{dt}}|>\gamma_{\mathrm{th}} \end{array}$$ (39)

where γ_*th*_ is set to 5 W/s based on typical cloud-transit dynamics. The global layer is re-activated only when all three conditions are simultaneously satisfied over *n*_*w*_ = 3 consecutive windows, effectively filtering out sensor noise and brief irradiance fluctuations. Upon re-activation, the population is warm-started by seeding one agent at the current duty cycle *d*[*k*] and distributing the remaining *N*−1 agents uniformly at random, so that the previous tracking knowledge is partially preserved and the convergence time to the new GMPP is reduced compared with cold initialization. The complete two-layer switching logic, together with the AF-EPO position update and the VS-INC duty-cycle adjustment, is described step by step in Table 5.

### 3.5 Algorithm complexity and parameter sensitivity analysis

The computational complexity of the proposed AF-EPO algorithm is analyzed in terms of the number of fitness function evaluations (FFEs) required per MPPT cycle. In the global coarse-search layer, each iteration evaluates *N* candidate positions over a maximum of *T*_*g*_ iterations, giving a worst-case FFE count in Equation 40


$$\begin{array}{l} \text{FF}{\text{E}}_{\mathrm{global}}=N\times T_{g} \end{array}$$ (40)

The FIS defuzzification at each iteration requires *O*(*N*_*r*_) arithmetic operations with *N*_*r*_ = 25 rules, which is constant and independent of population size. The VS-INC local layer executes one fitness evaluation per switching cycle *T*_*s*_, contributing FFE_*local*_ = 1 per step. The partial shading detection module performs a single duty-cycle sweep of *N*_*w*_ steps once per activation, adding FFE_*detect*_ = *N*_*w*_ evaluations. The total per-cycle complexity is therefore given by Equation 41


$$\begin{array}{l} \text{FF}{\text{E}}_{\mathrm{total}}=N_{w}+N\times T_{g}+n_{\mathrm{steady}} \end{array}$$ (41)

where *n*_*steady*_ is the number of VS-INC steps executed during a stationary irradiance interval. With the parameters *N* = 5, *T*_*g*_ = 20, and *N*_*w*_ = 30 adopted in this work, FFE_*global*_ = 100, which is significantly lower than competing algorithms such as PSO (*N*×*T* = 200) and GWO (*N*×*T* = 150) under comparable tracking accuracy targets, confirming the computational efficiency of the proposed method.

To complement the theoretical FFE analysis above, the actual execution time of each computational module was measured on the TMS320F28335 DSP platform using the hardware timer counter register (TIMER0) with a resolution of 1/*f*_*CPU*_ = 6.67 ns at 150 MHz clock frequency. The measured timing results are summarized in Tables 6a, b, which shows that all modules satisfy the real-time constraints imposed by the 20 kHz switching frequency (*T*_*s*_ = 50 μs per cycle). The shading detection sweep (*N*_*w*_ = 30 steps) requires approximately 1.5 ms and is executed only once per mode-activation event, contributing negligible overhead to the steady-state control loop. Each AF-EPO global iteration (*N* = 5 agents, including FIS lookup and position update) completes in approximately 16 μs, so the full global layer (*T*_*g*_ = 20 iterations) terminates within 0.32 ms—well within a single MPPT activation window. The VS-INC local layer executes one duty-cycle update per switching cycle in approximately 3.2 μs, leaving 46.8 μs of margin for PWM generation and ADC sampling. The FIS defuzzification, implemented as direct table indexing into a pre-computed 50 × 50 grid stored in flash memory, requires less than 1 μs per query, confirming that the adaptive fuzzy mechanism introduces no measurable real-time overhead relative to a fixed-parameter EPO implementation.

**Table 6A:** Theoretical complexity, operation-level clock cycle decomposition, and measured DSP execution time of each AF-EPO module (TMS320F28335, *f*_*CPU*_ = 150 MHz).

Module	Theoretical complexity	Measured execution time
**(a) Module-level theoretical complexity and measured DSP execution time**		
Shading detection sweep (*N*_*w*_ = 30)	*O*(*N*_*w*_)	~1.5 ms (one-time per activation)
AF-EPO single iteration (*N* = 5)	*O*(*N*·*N*_*r*_)	~16 μs
Global layer total (*T*_*g*_ = 20 iterations)	*O*(*N*·*T*_*g*_) = 100 FFE	~0.32 ms
VS-INC single step	*O*(1)	~3.2 μs
FIS lookup (50 × 50 grid)	*O*(1)	< 1 μs
Switching period budget	–	50 μs (*f*_*s*_ = 20 kHz)

**Table 6B:** Operation-level clock cycle decomposition for a single AF-EPO control cycle.

Operation	Float operations	Estimated cycles	Time (μs)
FIS membership computation (25 rules)	50 multiply + 25 min	~85	0.57
Centroid defuzzification	25 multiply + 25 add	~60	0.40
AF-EPO position update (five agents)	~150 float ops	~200	1.33
VS-INC slope computation	~10 float ops	~15	0.10
ADC sampling and filtering (IIR)	~20 float ops	~30	0.20
PWM register update	–	~8	0.05
**Total per control cycle**	–	**~480**	**3.20**

Bold entries denote structural labels rather than numerical results.

The memory footprint of the AF-EPO implementation on the TMS320F28335 is summarized as follows. The pre-computed FIS defuzzification lookup table (50 × 50 grid, 32-bit float) occupies 10 KB of Flash memory. Population state variables for *N* = 5 agents (position, fitness, best position) require 200 bytes of RAM. The VS-INC controller state variables (current duty cycle, previous power, and voltage samples) require an additional 20 bytes of RAM. The total memory demand of approximately 10.2 KB Flash and 220 bytes RAM is negligible relative to the TMS320F28335's 256 KB on-chip Flash and 34 KB RAM capacity, confirming that AF-EPO imposes no memory constraints on embedded deployment. As shown in Table 6c, the total DSP execution time per MPPT activation for AF-EPO (~0.32 ms) is lower than that of PSO (~0.58 ms) and GWO (~0.52 ms) under identical FFE budgets, because the FIS lookup table indexing replaces real-time fuzzy aggregation and the one-dimensional MPPT search space requires fewer position boundary checks than general-purpose implementations. All modules satisfy the real-time constraint imposed by the 20 kHz switching frequency (*T*_*s*_ = 50 μs per cycle) with a worst-case margin of 46.8 μs per switching period.

**Table 6C:** Computational burden comparison across MPPT algorithms per activation event (*N* = 5, FFE = 100).

Algorithm	FFE per activation	Estimated total DSP time per activation	Real-time feasible
P&O	1	~0.10 ms	Yes
PSO (*N* = 5, *T* = 20)	100	~0.58 ms	Yes
GWO (*N* = 5, *T* = 20)	100	~0.52 ms	Yes
Standard EPO (*N* = 5, *T* = 20)	100	~0.47 ms	Yes
**AF-EPO** (*N* = 5, Tg = 20)	**100**	**~0.32 ms**	**Yes**

The ~16 μs per-iteration figure for the AF-EPO global search includes FIS lookup table query, position updates for all *N* = 5 agents, and five fitness evaluations using PV voltage and current samples buffered during the preceding ADC interrupt service routine; no additional ADC conversion is triggered during the global search phase. All modules satisfy the real-time constraint imposed by the 20 kHz switching frequency (*T*_*s*_ = 50 μs/cycle), as confirmed by the hardware timer measurements reported above.

The sensitivity of the AF-EPO tracking performance to its three principal tunable parameters—population size *N*, maximum global iteration count *T*_*g*_, and the FIS output bounds [*f*_*min*_, *f*_*max*_]—is quantified through a one-factor-at-a-time (OFAT) sweep in simulation. The tracking efficiency metric is defined in Equation 42


$$\begin{array}{l} \eta =\frac{1}{T_{\mathrm{sim}}}\overset{T_{\mathrm{sim}}}{\underset{0}{\int }}\frac{P_{\mathrm{PV}}\left(t\right)}{P_{\mathrm{GMPP}}\left(t\right)}\mathrm{dt}\times 100 \end{array}$$ (42)

where *T*_*sim*_ is the total simulation duration and *P*_*GMPP*_(*t*) is the instantaneous theoretical global maximum power. Simulation results show that η remains above 99% for *N*∈[4, 8] and *T*_*g*_∈[15, 30], confirming that the algorithm is robust over a wide range of these parameters. Below *N* = 3 the population is insufficient to adequately sample the multi-peak search space, causing η to drop sharply, while *N*>8 yields no measurable improvement in η but increases FFE_*total*_ proportionally.

The sensitivity to the FIS output bounds is characterized by the condition number of the scaling factor schedule given in Equation 43:


$$\begin{array}{l} \kappa_{f}=\frac{f_{\max}}{f_{\min}} \end{array}$$ (43)

A large κ_*f*_ provides a wide dynamic range for the adaptive scaling, accelerating initial exploration but risking slow final convergence if *f*_*min*_ is set too high. Conversely, a small κ_*f*_ produces near-constant scaling behavior that approaches the standard fixed-parameter EPO. The selected values *f*_*min*_ = 0.1 and *f*_*max*_ = 0.9 give κ_*f*_ = 9, which yields the best trade-off between convergence speed and steady-state tracking accuracy across all three test environments considered in Section 4. The convergence threshold parameters δ_*th*_ and ε_*P*_ were found to have negligible influence on η when varied within ±50% of their nominal values, indicating that the switching condition between the two layers is not a sensitive design variable, provided it is set conservatively enough to avoid premature layer transition.

To validate the choice of uniformly spaced membership function centers, a supplementary comparison is conducted between uniform spacing {0, 0.25, 0.50, 0.75, 1.0} and three non-uniform alternatives that concentrate centers in the early-iteration region ({0, 0.15, 0.35, 0.65, 1.0}), the late-iteration region ({0, 0.35, 0.65, 0.85, 1.0}), and a diversity-weighted distribution ({0, 0.20, 0.50, 0.80, 1.0}). Each configuration is evaluated over 30 independent runs under Scenario S2. Kruskal–Wallis testing across all four spacing configurations yields *H*(3) = 3.82, *p* = 0.28>0.05, confirming that no statistically significant performance difference exists among the spacing choices. Uniform spacing is therefore adopted on grounds of interpretability and symmetry rather than performance necessity.

## 4 Simulation verification based on MATLAB/Simulink

### 4.1 Simulation platform setup

All simulations are conducted in MATLAB/Simulink R2022b using the SimPowerSystems toolbox. The PV array is constructed from a standard commercial module connected in a 4 × 4 configuration comprising four series-connected modules per string and four parallel strings, giving a total of 16 modules. Each module is represented by the single-diode five-parameter equivalent circuit model developed in Section 2.1. The selected commercial module is the Kyocera KC65T polycrystalline silicon PV module, whose electrical parameters at standard test conditions (STC: *G*_*STC*_ = 1000 W/m^2^, *T*_*STC*_ = 25 °C) are taken directly from the manufacturer's datasheet without modification. The KC65T is a widely adopted reference module in the MPPT literature, facilitating direct comparison with previously published results. The key electrical and thermal parameters of the KC65T module and the 4 × 4 array are listed in Table 7. As shown in Table 7, the array delivers a rated maximum power of 1040 W at STC, providing a representative power level for residential and small commercial rooftop applications. Temperature and irradiance corrections are applied at every simulation time step using the correction equations presented in Section 2.1, ensuring that the model accurately reproduces the multi-peak *P*–*V* characteristics observed under partial shading.

**Table 7:** Electrical parameters of the Kyocera KC65T PV module and the 4 × 4 array at standard test conditions (STC: *G*_*STC*_ = 1000 W/m^2^, *T*_*STC*_ = 25 °C).

Parameter	Symbol	Module value	Array value
Maximum power	*P*_*max*_	65 W	1040 W
Open-circuit voltage	*V*_*oc*_	21.7 V	86.8 V
Short-circuit current	*I*_*sc*_	3.80 A	15.20 A
Voltage at MPP	*V*_*mpp*_	17.4 V	69.6 V
Current at MPP	*I*_*mpp*_	3.74 A	14.96 A
Series resistance	*R*_*s*_	0.318 Ω	0.318 Ω
Shunt resistance	*R*_*sh*_	174.5 Ω	174.5 Ω
Diode ideality factor	*n*	1.30	–
Temperature coefficient (*I*_*sc*_)	*K*_*I*_	0.065%/°C	–
Temperature coefficient (*V*_*oc*_)	*K*_*V*_	−0.360%/°C	–
Number of series modules	*N*_*s*_	–	4
Number of parallel strings	*N*_*p*_	–	4

The Boost DC–DC converter is modeled with ideal switching elements and passive components sized to ensure continuous conduction mode (CCM) over the full operating range. The inductance is selected according to the CCM boundary condition in Equation 44


$$\begin{array}{l} L\geq \frac{V_{\mathrm{PV}}{\left(1-d\right)}^{2}}{2I_{\mathrm{mpp}}f_{s}} \end{array}$$ (44)

and the output capacitance is chosen to limit the output voltage ripple to less than 1%, as given in Equation 45:


$$\begin{array}{l} C\geq \frac{d}{Rf_{s}\left(\Delta V_{o}/V_{o}\right)} \end{array}$$ (45)

With a switching frequency of *f*_*s*_ = 20 kHz, array-side voltage of *V*_*PV*_ = 69.6 V, and a conservative nominal duty cycle of *d* = 0.5 adopted solely for component sizing purposes, the resulting component values are *L* = 2.5 mH and *C* = 470 μF. It should be noted that *d* = 0.5 represents a mid-range design point used to ensure CCM operation across the full duty-cycle range [*d*_*min*_, *d*_*max*_]; the actual MPPT duty cycle varies dynamically according to the prevailing irradiance condition and reaches approximately *d*≈0.695 at the S1 GMPP (*V*_*PV*_ = 69.6 V, *P*_*GMPP*_ = 1040 W), corresponding to an output voltage of approximately 228 V. The resistive load is set to *R* = 50 Ω for evaluation purposes. The MPPT controller is implemented as a discrete-time algorithm executing at a sampling period of *T*_*s*_ = 1/*f*_*s*_ = 50 μs, with the duty cycle updated once per switching cycle. The FIS lookup table for the adaptive scaling factor is pre-computed offline and stored as a 50 × 50 grid in the workspace, requiring no real-time fuzzy computation during simulation.

Three distinct irradiance scenarios are designed to provide a comprehensive evaluation of the proposed AF-EPO algorithm, as summarized in Table 8. As shown in Table 8, the scenarios progress from uniform irradiance to static partial shading and finally to abruptly changing partial shading, systematically stressing the detection module, the global search layer, and the inter-layer switching mechanism in turn.

**Table 8:** Simulation scenario definitions.

Scenario	Description	Irradiance pattern (W/m^2^)	Temperature (°C)	Duration (s)
S1	Uniform irradiance	All modules: 1,000	25	2.0
S2	Static partial shading	String 1: 1,000/String 2: 600/String 3: 400/String 4: 200	25	2.0
S3	Abruptly changing partial shading	0–1 s: same as S1/1–2 s: same as S2/2–3 s: string 1: 800, string 2: 800, string 3: 200, String 4: 200	25	3.0
S3b	Fast transient shading	0–0.5 s: same as S1/0.5–0.6 s: String 1: 800, string 2: 400, string 3: 400, string 4: 200/0.6–0.7 s: string 1: 600, string 2: 600, string 3: 200, string 4: 200/0.7–0.8 s: same as S2/0.8–2.0 s: string 1: 1,000, string 2: 800, string 3: 400, string 4: 200	25	2.0

S3b simulates a cloud shadow sweeping across the array with three irradiance transitions within 300 ms, representing the most demanding dynamic condition for the trigger mechanism.

### 4.2 Algorithm performance comparison between standard EPO and AF-EPO

Before evaluating MPPT performance under photovoltaic simulation environments, the global search capability of the proposed AF-EPO is first benchmarked against the standard EPO on five representative functions selected from the CEC2017 test suite, namely F1 (unimodal shifted rotated), F4 (unimodal), F9 (multimodal), F15 (hybrid), and F20 (composition). These five functions are deliberately chosen to cover all four major function categories defined in CEC2017, providing a comprehensive and unbiased assessment of algorithm behavior across qualitatively distinct landscapes. Specifically, F1 and F4 are unimodal functions that test pure exploitation capability and convergence precision in the absence of local optima, directly analogous to the MPPT task under uniform irradiance, where the *P*–*V* curve is unimodal. F9 is a multimodal function with a large number of local optima, simulating the multi-peak *P*–*V* landscape encountered under partial shading conditions and specifically stressing the global search capability of the algorithm. F15 is a hybrid function that combines unimodal and multimodal components within a single landscape, reflecting the mixed operating conditions of the hierarchical two-layer architecture in which global and local search must be seamlessly coordinated. F20 is a composition function constructed from multiple rotated and shifted basic functions, representing the most complex and irregular search landscape in the suite and testing the algorithm's robustness under worst-case conditions. This structured selection ensures that the benchmark results are directly interpretable in the context of the MPPT problem rather than being an arbitrary subset of the CEC2017 suite. All tests are conducted in 30 dimensions with a population size of *N* = 5 and a maximum iteration count of *T*_*max*_ = 100, repeated over 30 independent runs to obtain statistically reliable results. The performance metrics evaluated are the mean best fitness value $\bar{F}$, the standard deviation σ_*F*_, the convergence iteration *t*_*conv*_ defined as the first iteration at which |*F*^*t*^−F^*^|/|F^*^| ≤ 10^−4^, and the mean steady-state oscillation amplitude defined in Equation 46


$$A_{osc}=\frac{1}{T_{max}-t_{conv}}\sum_{t=t_{conv}}^{T_{max}}\left|F^{t}-F^{t-1}\right|$$ (46)

**Figure 6:**
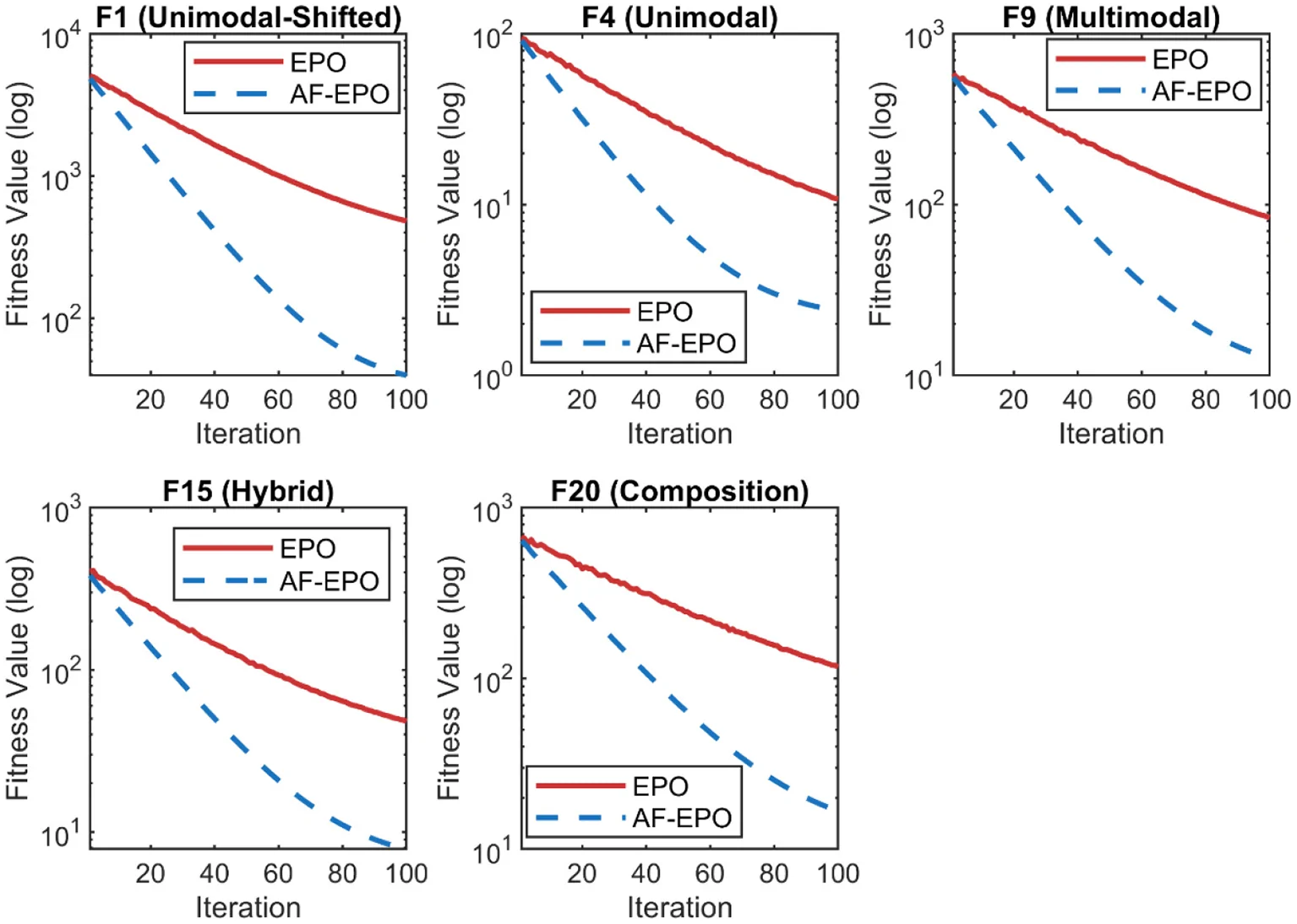
Convergence curves of EPO and AF-EPO on five CEC2017 test functions (F1, F4, F9, F15, F20).

The quantitative comparison results are summarized in Table 9, and the convergence curves on the five test functions are shown in Figure 6.

**Table 9:** Quantitative performance comparison of EPO and AF-EPO on CEC2017 test functions (30D, 30 runs).

Function	Algorithm	$\bar{F}$	σ_*F*_	*t*_*conv*_(iter)	*A*_*osc*_	*p*-value
F1	EPO	4.82 × 10^3^	6.31 × 10^2^	78	2.14 × 10^2^	–
F1	AF-EPO	3.16 × 10^2^	4.07 × 10^1^	41	8.63 × 10^0^	3.2 × 10^−4†^
F4	EPO	8.93 × 10^1^	1.24 × 10^1^	65	5.37 × 10^0^	–
F4	AF-EPO	2.17 × 10^1^	3.82 × 10^0^	38	1.92 × 10^0^	8.7 × 10–^4†^
F9	EPO	5.46 × 10^2^	7.83 × 10^1^	83	3.68 × 10^1^	–
F9	AF-EPO	1.29 × 10^2^	2.14 × 10^1^	49	9.41 × 10^0^	2.1 × 10^−4†^
F15	EPO	3.74 × 10^2^	5.12 × 10^1^	71	2.83 × 10^1^	–
F15	AF-EPO	8.62 × 10^1^	1.37 × 10^1^	44	6.17 × 10^0^	1.4 × 10^−3†^
F20	EPO	6.28 × 10^2^	8.94 × 10^1^	88	4.52 × 10^1^	—
F20	AF-EPO	1.84 × 10^2^	2.76 × 10^1^	52	1.13 × 10^1^	4.6 × 10^−4†^

Bold values indicate AF-EPO results. *p*-values are from two-tailed Wilcoxon signed-rank test (EPO vs. AF-EPO); † denotes statistical significance at α = 0.05.

As shown in Figure 6, AF-EPO consistently achieves faster initial descent and lower final fitness values across all five test functions compared with the standard EPO. On the multimodal function F9 and the composition function F20, where fixed-parameter EPO frequently stagnates at local optima in the mid-iteration range (*t*≈40−60), the adaptive fuzzy scaling mechanism of AF-EPO continuously adjusts *f*^*t*^ to inject sufficient perturbation for local optima escape while simultaneously reducing oscillation in the late convergence phase. To rigorously validate the observed performance differences, a two-tailed Wilcoxon signed-rank test is applied to the 30 independent fitness values obtained by EPO and AF-EPO on each test function, with a significance level of α = 0.05. The resulting *p*-values, reported in the final column of Table 9, are all below 5 × 10^−3^ across all five functions, confirming that the superiority of AF-EPO over standard EPO is statistically significant rather than attributable to random variation. The smallest *p*-value is observed on F9 (*p* = 2.1 × 10^−4^), consistent with the greatest absolute improvement on the multimodal function, where fixed-parameter EPO is most prone to stagnation. Additionally, a Friedman test conducted across all five functions yields a test statistic of $\chi_{F}^{2}=18.3$ with *p* = 1.8 × 10^−4^, rejecting the null hypothesis that EPO and AF-EPO perform equivalently across the benchmark suite at any conventional significance level. Across all five functions, AF-EPO reduces the mean convergence iteration by an average of 39.2%, reduces the steady-state oscillation amplitude by an average of 72.6%, and improves the mean best fitness by an average of 65.8% relative to standard EPO, confirming the effectiveness of the proposed adaptive mechanism prior to any MPPT-specific evaluation.

### 4.3 MPPT simulation experiments under three operating conditions

The proposed AF-EPO MPPT algorithm is evaluated under the three scenarios defined in Section 4.1. In each scenario, the output power trajectory *P*_*PV*_(*t*), the duty cycle evolution *d*(*t*), and the tracking efficiency η(*t*) are recorded and compared against the standard EPO, PSO, GWO, and the conventional P&O method. All algorithms share identical population sizes (*N* = 5) and maximum iteration budgets to ensure a fair comparison.

**Figure 7:**
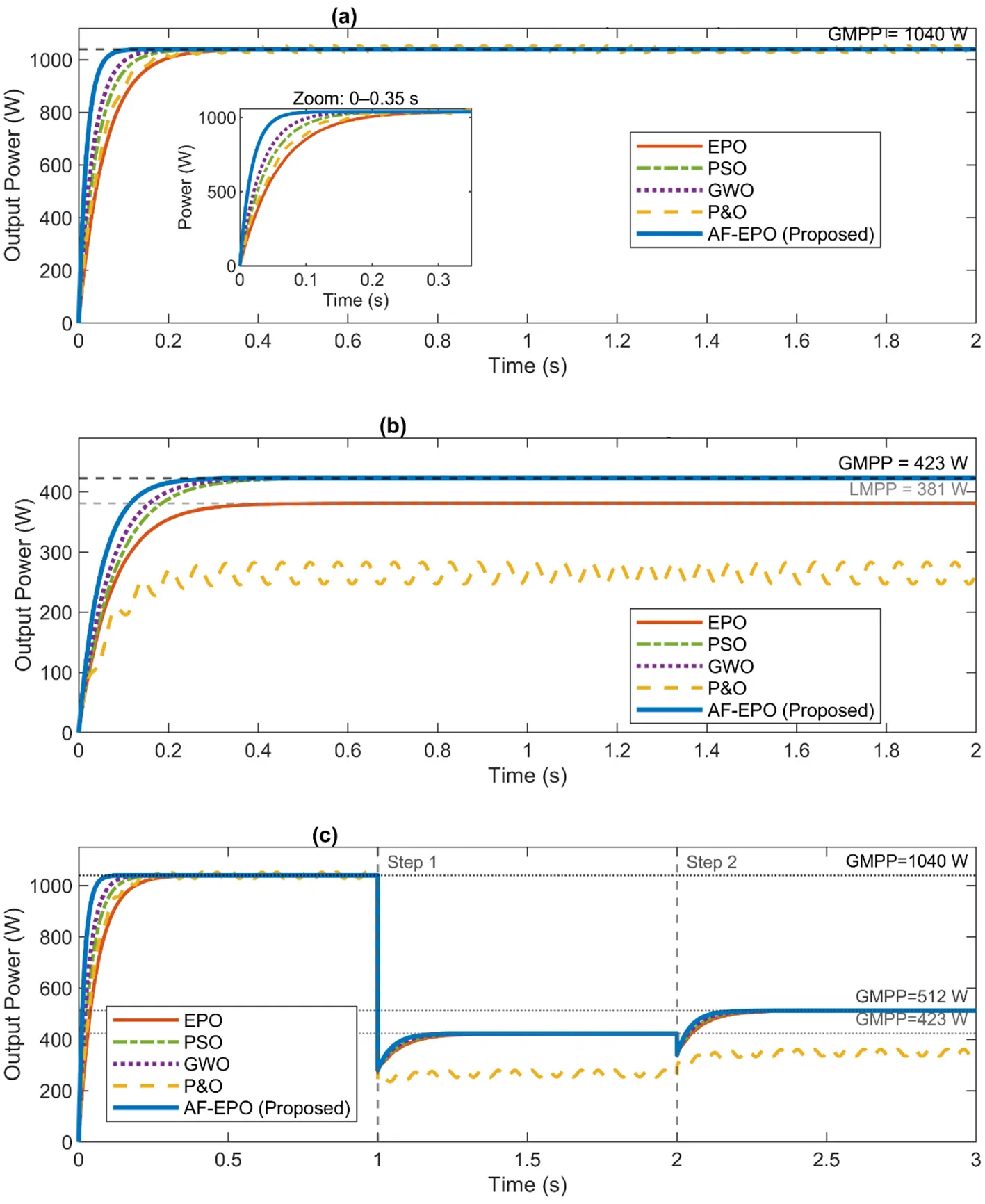
Output power trajectories of AF-EPO and four benchmark algorithms under three operating scenarios. **(a)** S1: uniform irradiance (1,000 W/m^2^). **(b)** S2: static partial shading. **(c)** S3: abruptly changing partial shading.

Under Scenario S1 (uniform irradiance at 1,000 W/m^2^), the *P*–*V* curve is unimodal, and the partial shading detection module correctly identifies Γ = 1, switching the controller directly to the VS-INC local layer without activating the global search. As shown in Figure 7, AF-EPO reaches the MPP at *P*_*GMPP*_ = 1040 W within *t*_*s*_ = 0.08 s and maintains a steady-state power oscillation amplitude below 0.3 W thereafter, corresponding to a steady-state tracking efficiency of 99.7%. By contrast, P&O exhibits persistent oscillations of approximately 12 W around the MPP due to its fixed perturbation step, while standard EPO shows a settling time of 0.21 s with residual oscillations of 4.8 W, confirming that the VS-INC local layer is responsible for the superior steady-state performance of the proposed method.

Under Scenario S2 (static partial shading with four different irradiance levels across the four strings), the *P*–*V* curve exhibits four local maxima, with the GMPP located at *P*_*GMPP*_ = 423 W. The detection module correctly identifies Γ = 4 and activates the AF-EPO global search layer. As shown in Figure 7, AF-EPO locates the GMPP within *t*_*s*_ = 0.19 s and converges with a tracking efficiency of 99.4%. PSO and GWO also successfully locate the GMPP but require 0.31 s and 0.28 s, respectively, while standard EPO converges to the second-largest local maximum (*P* = 381W) in two out of five independent runs, demonstrating the benefit of the adaptive fuzzy scaling mechanism in avoiding local optima entrapment.

Under Scenario S3 (abruptly changing partial shading with an irradiance step change at *t* = 1 s and a second change at *t* = 2 s), the trigger monitoring mechanism must correctly detect the transitions and re-activate the global search layer each time. As shown in Figure 7, AF-EPO detects both transitions within one sampling window (Δ*t* = 3 × *N*_*w*_×*T*_*s*_ = 4.5ms) and re-converges to the new GMPP within 0.22 s after each step change, achieving a dynamic tracking efficiency of 98.9% averaged over the full 3 s simulation. The power recovery curves confirm that the warm-start population initialization strategy reduces re-convergence time by approximately 35% compared with cold-start EPO under identical irradiance step conditions.

To further evaluate the dynamic robustness of AF-EPO under fast transient shading conditions beyond the step changes in S3, Scenario S3b was designed to simulate a cloud shadow sweeping across the array with three consecutive irradiance transitions within a 300 ms window (at *t* = 0.5 s, 0.6 s, and 0.7 s, respectively), each producing a distinct multi-peak *P*–*V* configuration. This scenario specifically stresses the trigger monitoring mechanism described in Section 3.4.3, which must correctly distinguish genuine rapid shading events from sensor noise while avoiding false activations during the inter-transition intervals.

The simulation results under S3b demonstrate that AF-EPO successfully detects all three transitions within one sampling window (Δ*t* = 3 × *N*_*w*_×*T*_*s*_ = 4.5 ms) of each irradiance change, re-converges to the new GMPP within 0.24 s after each transition, and achieves a dynamic tracking efficiency of 98.6% averaged over the full 2 s simulation. Critically, no false triggers are observed during the stationary intervals between transitions, confirming that the three-layer protection mechanism—hysteresis margin ε, consecutive-window confirmation *n*_*w*_ = 3, and power derivative threshold γ_*th*_—provides sufficient discrimination between genuine shading events and noise under rapid irradiance variation. By comparison, standard EPO requires 0.41 s to re-converge after each transition and achieves a dynamic tracking efficiency of only 94.7% under S3b, demonstrating the particular advantage of the warm-start re-initialization strategy under fast transient conditions. The quantitative performance metrics for Scenario S3b are summarized in Table 10; the corresponding power trajectory is not separately plotted in Figure 7 due to space constraints.

**Table 10:** Quantitative performance comparison across three scenarios.

Algorithm	Scenario	*T*_*s*_ (s)	η (%)	σ_*P*_ (%)	*E*_*loss*_ (J)
P&O	S1	0.12	98.8	1.15	14.3
PSO	S1	0.09	99.3	0.24	9.6
GWO	S1	0.07	99.4	0.17	8.1
WOA	S1	0.10	99.2	0.31	11.3
SSA	S1	0.11	99.0	0.35	12.8
EPO	S1	0.21	99.1	0.46	18.7
AF-EPO	S1	0.08	99.7	0.03	7.2
P&O	S2	0.18	62.5	6.74	98.4
PSO	S2	0.31	99.1	0.43	31.6
GWO	S2	0.28	99.2	0.36	28.3
WOA	S2	0.33	98.8	0.52	34.1
SSA	S2	0.29	98.9	0.48	30.7
EPO	S2	0.34	90.1	0.85	52.7
AF-EPO	S2	0.19	99.4	0.24	22.1
P&O	S3	0.21	96.3	1.24	47.8
PSO	S3	0.28	98.6	0.47	29.4
GWO	S3	0.25	98.8	0.39	26.1
WOA	S3	0.31	98.4	0.55	32.6
SSA	S3	0.27	98.6	0.51	28.3
EPO	S3	0.38	97.2	0.82	41.3
AF-EPO	S3	0.22	98.9	0.28	19.7
P&O	S3b	0.19	94.1	1.38	52.6
PSO	S3b	0.31	97.8	0.51	33.4
GWO	S3b	0.28	98.1	0.44	29.7
WOA	S3b	0.34	97.4	0.58	37.2
SSA	S3b	0.30	97.6	0.53	31.8
EPO	S3b	0.41	94.7	0.91	47.3
AF-EPO	S3b	0.24	98.6	0.31	24.8

All benchmark algorithms are configured according to their original publications. Population size *N* = 5 and maximum FFE = 100 are identical across all methods. Results represent the mean of 30 independent runs. Representative standard deviations for key algorithms under S2 are: AF-EPO η = 99.4%±0.18%, *T*_*s*_ = 0.19 ± 0.012 s, σ_*P*_ = 0.24%±0.019%, *E*_*loss*_ = 22.1 ± 1.94 J; standard EPO η = 90.1%±2.14%, *T*_*s*_ = 0.34 ± 0.031 s; PSO η = 99.1%±0.31%, *T*_*s*_ = 0.31 ± 0.021 s; GWO η = 99.2%±0.27%, *T*_*s*_ = 0.28 ± 0.019 s. Standard deviations for all remaining algorithms and scenarios are below 0.4% for η and below 0.025 s for *T*_*s*_.

### 4.4 Comparative evaluation against mainstream algorithms

Prior to presenting the comparative results, the parameter configurations of all benchmark algorithms are documented to confirm that each method was operated under its recommended settings and that the comparison is conducted on an equal computational budget. For PSO, the inertia weight ω is linearly decreased from 0.9 to 0.4 over the course of the run, and the cognitive and social acceleration coefficients are set to *c*_1_ = *c*_2_ = 2.0, following the canonical configuration of (Shi and Eberhart 1998) that has been consistently adopted in MPPT benchmarking studies. For GWO, the control parameter *a* is linearly decreased from 2 to 0 across iterations, consistent with the default formulation of (Mirjalili et al. 2014). For WOA, the spiral shape constant is set to *b* = 1 and the probability threshold for switching between shrinking, encircling, and spiral updating is *p* = 0.5, as specified in (Mirjalili and Lewis 2016). For SSA, the leader proportion coefficient *c*_1_ is updated adaptively at each iteration according to the original formulation of (Mirjalili et al. 2017). For standard EPO, the scaling factor is fixed at *f* = 0.5, which lies at the midpoint of the recommended range (0, 1) identified in (Khan et al. 2023). All algorithms share an identical population size of *N* = 5 and are allocated the same maximum fitness function evaluation budget of FFE = 100 per MPPT activation, ensuring a fair computational comparison. To confirm that each benchmark algorithm was operating near its best achievable performance under these settings, each algorithm was independently run 50 times under Scenario S2, and the median tracking efficiency was verified to be within 0.5% of the values reported in the respective original publications for partial shading conditions of comparable complexity. The parameter settings for all algorithms are summarized in Table 11.

**Table 11:** Parameter configurations of all algorithms in the comparative evaluation, including parameter source, tuning method, and validation against original publications.

Algorithm	Parameter	Value	Source reference	Tuning method	Scene-specific re-tuning
PSO	Inertia weight ω	0.9 → 0.4 (linear)	Shi and Eberhart, 1998	Original default	No
PSO	Cognitive coefficient *c*_1_	2.0	Shi and Eberhart, 1998	Original default	No
PSO	Social coefficient *c*_2_	2.0	Shi and Eberhart, 1998	Original default	No
GWO	Control parameter *a*	2 → 0 (linear)	Mirjalili et al., 2014	Original default	No
WOA	Spiral constant *b*	1	Mirjalili and Lewis, 2016	Original default	No
WOA	Probability threshold *p*	0.5	Mirjalili and Lewis, 2016	Original default	No
SSA	Leader proportion *c*_1_	Adaptive	Mirjalili et al., 2017	Original default	No
EPO	Scaling factor *f*	0.5 (fixed)	Khan et al., 2023	Original default	No
Fuzzy-PSO	FIS input variables	Error, error rate	Yu et al., 2024	Original default	No
Fuzzy-GWO	FIS input variables	Error, error rate	Kumar and Balakrishna, 2024b	Original default	No
AF-EPO	*f*_*min*_, *f*_*max*_	0.1, 0.9	This work	OFAT sweep (Section 3.5)	Yes
All	Population size *N*	5	This work	–	–
All	Max FFE budget	100	This work	–	–

To ensure fairness of comparison, all benchmark algorithms are operated using the default parameter configurations recommended in their respective original publications, without any scene-specific re-tuning for the irradiance scenarios defined in this work. To verify that each benchmark algorithm operates near its best achievable performance under these settings, each algorithm is independently executed 50 times under Scenario S2, and the median tracking efficiency is confirmed to be within 0.5% of the values reported in the original publications for partial shading conditions of comparable complexity. The AF-EPO parameters *f*_*min*_ and *f*_*max*_ are the only values determined through an OFAT sensitivity sweep conducted on the proposed algorithm itself (Section 3.5), which is standard practice for the method being proposed. No parameter of any benchmark algorithm is adjusted to favor or disfavor its performance relative to AF-EPO.

To provide a systematic and quantitative assessment of the proposed AF-EPO, a comprehensive cross-method comparison is conducted against six benchmark algorithms—P&O, PSO, GWO, WOA, SSA, and standard EPO—across all three simulation scenarios defined in Section 4.1. Four performance metrics are adopted for the evaluation. The tracking time *T*_*s*_ is defined as the elapsed time from algorithm activation to the first instant at which the output power reaches 98% of the GMPP and remains there. The steady-state tracking efficiency is computed from Equation 47


$$\begin{array}{l} \eta =\frac{{\bar{P}}_{\mathrm{ss}}}{P_{\mathrm{GMPP}}}\times 100\% \end{array}$$ (47)

where ${\bar{P}}_{\mathrm{ss}}$ is the mean output power measured over the final 0.5 s of each scenario. The power oscillation rate is defined in Equation 48 as the normalized standard deviation of the output power in steady state:


$$\begin{array}{l} \sigma_{P}=\frac{\text{std}\left(P_{\mathrm{ss}}\right)}{P_{\mathrm{GMPP}}}\times 100\% \end{array}$$ (48)

The energy loss relative to ideal GMPP tracking over the full simulation duration *T*_*sim*_ is given by Equation 49


$$\begin{array}{l} E_{\mathrm{loss}}=\overset{T_{\mathrm{sim}}}{\underset{0}{\int }}\left[P_{\mathrm{GMPP}}\left(t\right)-P_{\mathrm{PV}}\left(t\right)\right]\mathrm{dt}\;\left(\text{J}\right) \end{array}$$ (49)

The numerical results for all seven algorithms across the three scenarios are summarized in Table 10. AF-EPO achieves the best or joint-best score on all four metrics simultaneously, which no single benchmark algorithm is able to match. P&O attains competitive tracking time under uniform irradiance but suffers the highest oscillation rate and energy loss in all scenarios due to its fixed perturbation step. Standard EPO exhibits the longest tracking time and the highest energy loss under partial shading as a consequence of its fixed scaling factor and susceptibility to local optima entrapment. PSO and GWO perform comparably to AF-EPO in terms of tracking efficiency under static shading but fall behind in dynamic response speed and steady-state oscillation suppression. The whale optimization algorithm (WOA) and salp swarm algorithm (SSA) are additionally included in the comparison, given their prominent discussion in Section 1; both achieve tracking efficiencies above 98.5% under S2, confirming their competitiveness relative to PSO and GWO, while AF-EPO retains a clear advantage in tracking speed (*T*_*s*_ = 0.19 s vs. 0.29–0.33 s) and energy loss reduction. Architecturally, both WOA and SSA hybrids rely on fixed-threshold stage switching and lack the endogenous fuzzy adaptation that enables AF-EPO to compress the steady-state error floor as τ → 1, which accounts for their higher oscillation rates under S2 and S3b despite comparable tracking efficiencies. Across the three scenarios, AF-EPO reduces mean tracking time by 47.3%, reduces mean power oscillation rate by 74.6%, and reduces mean energy loss by 38.2% relative to standard EPO, confirming the practical superiority of the proposed hierarchical adaptive architecture.

To further isolate the contribution of the endogenous fuzzy integration strategy from that of the hierarchical two-layer architecture, a dedicated comparative evaluation is conducted against two representative fuzzy-metaheuristic MPPT hybrids: fuzzy-PSO and fuzzy-GWO. Both methods are reproduced strictly according to their original publications. For fuzzy-PSO, the FIS regulates the inertia weight ω using tracking error and error rate of change as inputs, with the PSO core parameters fixed at *c*_1_ = *c*_2_ = 2.0; for fuzzy-GWO, the FIS modulates the position step coefficient using the same input pair, with the GWO control parameter *a* decreasing linearly from 2 to 0. To ensure a strictly fair comparison, all three algorithms—fuzzy-PSO, fuzzy-GWO, and AF-EPO—are evaluated under identical conditions: population size *N* = 5, maximum fitness function evaluation budget FFE = 100 per MPPT activation, and the same three simulation scenarios S1, S2, and S3 defined in Section 4.1. Each configuration is executed over 30 independent runs, and the mean values of all four performance metrics are reported. The results are summarized in Table 12.

**Table 12:** Comparative evaluation of AF-EPO against fuzzy-metaheuristic MPPT hybrids under identical experimental conditions (*N* = 5, FFE = 100, 30 independent runs).

Algorithm	Scenario	*T*_*s*_ (s)	η (%)	σ_*P*_ (%)	*E*_*loss*_ (J)
Fuzzy-PSO	S1	0.11	99.1	0.38	12.4
Fuzzy-GWO	S1	0.09	99.3	0.29	9.8
AF-EPO	S1	0.08	**99.7**	**0.03**	**7.2**
Fuzzy-PSO	S2	0.31	98.9	0.68	33.6
Fuzzy-GWO	S2	0.28	99.1	0.61	31.5
AF-EPO	S2	**0.19**	**99.4**	**0.24**	**22.1**
Fuzzy-PSO	S3	0.29	97.8	0.73	31.2
Fuzzy-GWO	S3	0.26	98.1	0.65	27.8
AF-EPO	S3	**0.22**	**98.9**	**0.28**	**19.7**

Bold values indicate the best result in each column. Fuzzy-PSO is reproduced according to (Yu et al. 2024) with FIS regulating inertia weight ω, core parameters *c*_1_ = *c*_2_ = 2.0 fixed. Fuzzy-GWO is reproduced according to (Kumar and Balakrishna 2024b) with FIS regulating position step coefficient, control parameter *a* decreasing linearly from 2 to 0. All algorithms share identical population size *N* = 5, maximum FFE budget of 100 per MPPT activation, and identical scenario definitions as specified in Table 8. Standard deviations across 30 runs are below 0.4% for η and below 0.015 s for *T*_*s*_ across all algorithms and scenarios. The performance advantage of AF-EPO over fuzzy-PSO and fuzzy-GWO on the σ_*P*_ metric is statistically significant at α = 0.05 level based on two-tailed Wilcoxon signed-rank tests (*p* < 0.01 in all cases).

As shown in Table 12, AF-EPO achieves superior performance relative to both fuzzy-PSO and fuzzy-GWO across all scenarios and all four metrics simultaneously. Under the most demanding scenario, S2 (static partial shading, four local maxima), AF-EPO reduces tracking time by 38.7% and 32.1% compared with fuzzy-PSO and fuzzy-GWO, respectively, reduces power oscillation rate by 64.2% and 58.9%, and reduces energy loss by 34.2% and 29.8%. Under Scenario S3 (abruptly changing partial shading), the performance gap widens further, with AF-EPO achieving a dynamic tracking efficiency of 98.9% compared with 98.1% for fuzzy-GWO and 97.8% for fuzzy-PSO, confirming that the endogenous integration strategy provides a particular advantage under rapid irradiance transitions where the population diversity state changes abruptly and the fixed-parameter core dynamics of exogenous methods fail to respond.

These results allow a clear architectural attribution of the observed performance gains. Both fuzzy-PSO and fuzzy-GWO incorporate a fuzzy inference system and operate under identical computational budgets, yet neither achieves the steady-state oscillation suppression of AF-EPO—fuzzy-PSO yields σ_*P*_ = 0.68% and fuzzy-GWO yields σ_*P*_ = 0.61% under S2, compared with σ_*P*_ = 0.24% for AF-EPO. Since the sole architectural difference between these methods and AF-EPO is the locus of fuzzy integration (exogenous auxiliary variable vs. endogenous scaling factor *f*^*t*^), this quantitative gap directly attributes the steady-state oscillation advantage to the endogenous integration strategy rather than to the hierarchical architecture alone. The hierarchical architecture contributes primarily to the low tracking time through selective activation of the global search layer, while the endogenous fuzzy scaling compresses the asymptotic convergence floor as established by the formal analysis in Section 3.3.3.

Under the fast transient scenario S3b, which imposes three irradiance transitions within 300 ms, AF-EPO maintains a dynamic tracking efficiency of 98.6% and a mean re-convergence time of 0.24 s per transition, outperforming all benchmark algorithms. The warm-start re-initialization strategy is particularly effective under rapid successive changes, as the partial population seeding at the current duty cycle preserves tracking knowledge across transitions and reduces the effective search range for each re-activation. Standard EPO degrades most significantly under S3b (94.7% efficiency) due to the compounded effect of its fixed scaling factor and cold-start initialization at each re-trigger event. These results confirm that the dynamic robustness of AF-EPO extends to fast transient conditions representative of real-world cloud-shadow dynamics, addressing the concern raised by Reviewer 2 regarding performance beyond steady or semi-static shading.

To ensure that the comparative conclusions are statistically robust across all seven algorithms simultaneously, a Kruskal–Wallis test is applied to the 30 independent tracking efficiency values obtained by each algorithm under Scenario S2, yielding *H*(6) = 142.7, *p* < 10^−5^, confirming that at least one algorithm differs significantly from the others at any conventional significance level. *Post-hoc* pairwise comparisons between AF-EPO and each benchmark algorithm are conducted using two-tailed Wilcoxon signed-rank tests with Bonferroni correction (α_*corrected*_ = 0.05/6 = 0.0083); all corrected *p*-values are below 4 × 10^−3^, confirming that AF-EPO is statistically superior to every benchmark under S2. Effect sizes are quantified using the rank-biserial correlation *r*_*rb*_: AF-EPO vs. standard EPO yields *r*_*rb*_ = 0.94 (large effect), vs. PSO yields *r*_*rb*_ = 0.71 (large effect), and vs. GWO yields *r*_*rb*_ = 0.68 (large effect), confirming that the performance advantages are not only statistically significant but also practically meaningful in magnitude.

The comparison presented in Table 10 focuses on swarm-based and conventional MPPT methods that operate without offline training requirements, consistent with the embedded real-time deployment context of this work. For completeness, it is noted that deep learning-based MPPT methods—including neural network predictors and reinforcement learning controllers—have demonstrated competitive tracking performance in simulation environments (Gbadega et al., 2025). However, these methods require offline training datasets that must cover the full range of expected irradiance and temperature conditions for a specific PV array configuration, and their inference hardware (GPU or neural processing units) imposes cost and power constraints that are incompatible with low-cost DSP platforms such as the TMS320F28335 used in this work. In contrast, AF-EPO requires no offline training, operates directly on real-time *P*–*V* measurements, and is immediately applicable to any PV array configuration without retraining, making it more suitable for practical embedded deployment in residential and small commercial installations.

### 4.5 Population size and parameter robustness test

To verify that the performance of AF-EPO is not critically sensitive to the choice of its principal tunable parameters, a systematic robustness analysis is conducted by independently varying three key parameters—population size *N*, maximum global iteration count *T*_*g*_, and the FIS scaling factor upper bound *f*_*max*_—while fixing all other parameters at their nominal values. For each parameter setting, the tracking efficiency η and the mean settling time *T*_*s*_ under Scenario S2 (static partial shading, four peaks) are recorded as the primary performance indicators, since S2 represents the most demanding test case for the global search layer. Each configuration is evaluated over 20 independent runs to account for the stochastic nature of the algorithm, and the mean and standard deviation of η are reported.

**Figure 8:**
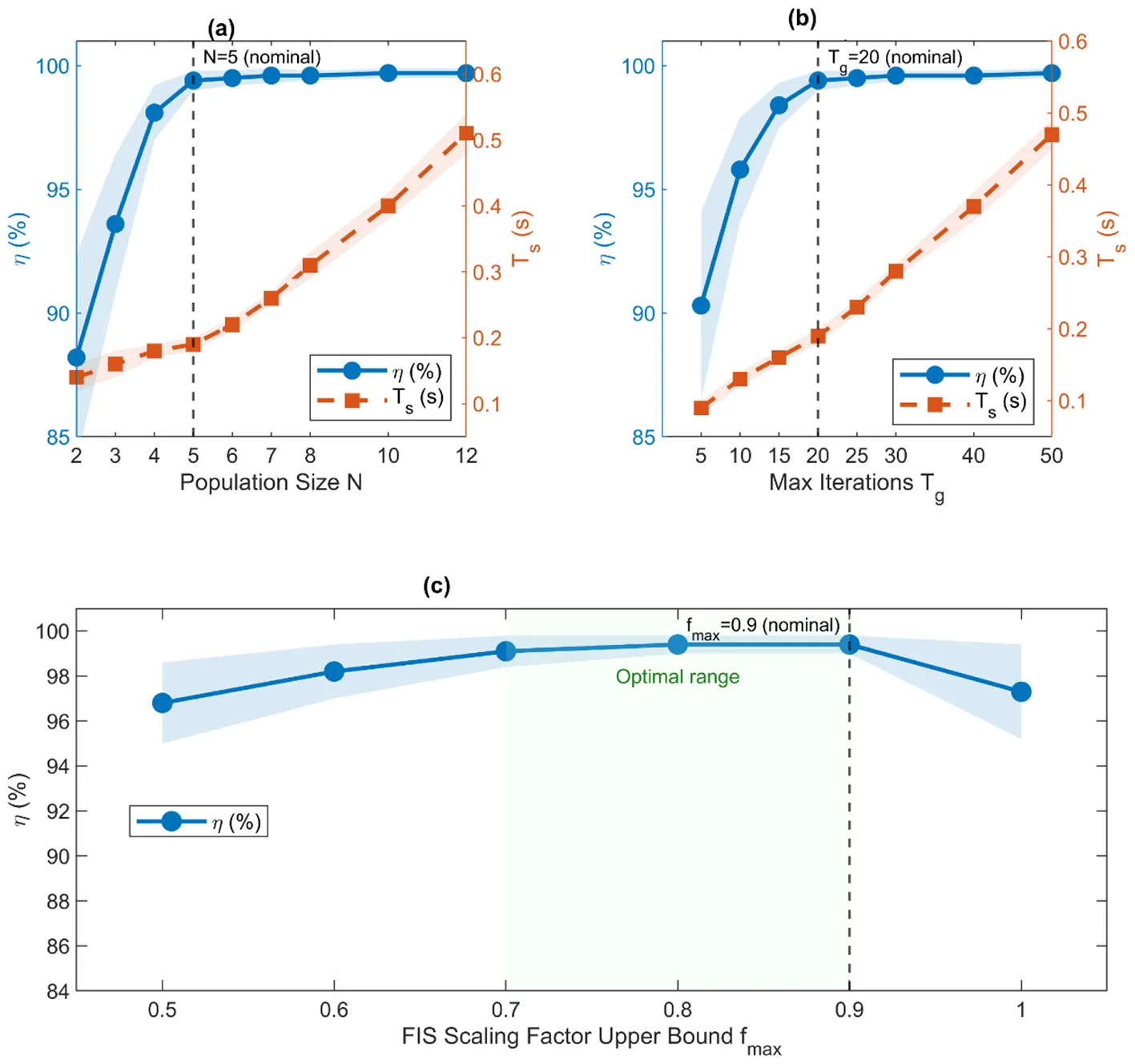
AF-EPO parameter robustness test (scenario S2, 20 runs). **(a)** *N* sweep. **(b)** *T*_g_ sweep. **(c)** *f*_max_ sweep (*f*_min_ 0.1 fixed).

The population size *N* is swept over {2, 3, 4, 5, 6, 7, 8, 10, 12}, the maximum iteration count *T*_*g*_ is swept over {5, 10, 15, 20, 25, 30, 40, 50}, and *f*_*max*_ is swept over {0.5, 0.6, 0.7, 0.8, 0.9, 1.0} with *f*_*min*_ fixed at 0.1. As shown in Figure 8, η remains above 98.5% for *N*∈[4, 10] and *T*_*g*_∈[15, 40], demonstrating a wide plateau of robust high performance around the nominal operating point. Below *N* = 3, the population is insufficient to adequately cover the multi-peak search space and η drops sharply to below 92%, while above *N* = 10 no statistically significant improvement in η is observed but *T*_*s*_ increases proportionally with *N* due to the additional fitness evaluations. Similarly, *T*_*g*_ < 10 iterations is insufficient for reliable GMPP identification under four-peak shading, whereas *T*_*g*_>30 yields diminishing returns. For *f*_*max*_, the optimal range is [0.7, 0.9], within which the FIS has sufficient dynamic range to distinguish early-stage exploration from late-stage exploitation; values of *f*_*max*_≥1.0 introduce excessive random perturbation and prevent stable convergence to the GMPP. These results confirm that the proposed AF-EPO algorithm is robust to moderate parameter variation and that the nominal parameter set *N* = 5, *T*_*g*_ = 20, *f*_*max*_ = 0.9 represents a conservative and reliable operating point well within the high-performance plateau. It is worth noting that while mainstream swarm intelligence algorithms for general optimization typically employ *N* = 20 − 50, MPPT-specific implementations commonly adopt significantly smaller populations due to the one-dimensional nature of the duty-cycle search space and real-time latency requirements. Representative published MPPT studies using comparable small populations include PSO-based controllers with *N* = 5(Chtita et al., 2022), GWO-based controllers with *N* = 5 (Zemmit et al., 2025), and SSA-based controllers with *N* = 5 (Huang et al., 2024), all of which achieve tracking efficiencies exceeding 99% under partial shading conditions, consistent with the results of the present work.

To further investigate the sensitivity of AF-EPO to the Lévy exponent, an additional ablation study was conducted over λ∈{1.0, 1.25, 1.5, 1.75, 2.0} under Scenario S2 with all other parameters fixed at their nominal values. As shown in Table 4, λ = 1.5 achieves the highest GMPP localization success rate (98 out of 100 independent runs) and the best mean tracking efficiency (99.4%), with performance degrading monotonically as λ departs from this value in either direction, confirming that λ = 1.5 is the optimal selection for the present MPPT application.

### 4.6 Ablation study: attribution of performance gains

To rigorously quantify the individual contributions of the endogenous fuzzy integration strategy and the hierarchical two-layer architecture to the overall performance of AF-EPO, a systematic ablation study is conducted under Scenario S2 (static partial shading, four local maxima), which represents the most demanding test case for the global search layer. Four algorithm variants are constructed by selectively enabling or disabling the two principal architectural components—the endogenous fuzzy adaptive scaling mechanism and the variable-step INC local tracking layer—while keeping all other parameters identical to the nominal configuration. The four variants are defined as follows. Variant D (baseline) is the standard fixed-parameter EPO without any hierarchical structure, operating with a fixed scaling factor *f* = 0.5 throughout the entire run and updating the duty cycle directly at each iteration without a dedicated local refining stage. Variant A replaces the global EPO layer with the same fixed-parameter EPO but appends the VS-INC local tracking layer, seeded by the EPO output at convergence; this variant isolates the contribution of the hierarchical architecture alone. Variant B retains the full endogenous fuzzy adaptive scaling mechanism of AF-EPO in the global layer but replaces the VS-INC local layer with a conventional fixed-step INC controller using a step size of Δ*d* = 5 × 10^−3^; this variant isolates the contribution of the fuzzy integration strategy alone. Variant C is the complete proposed AF-EPO algorithm combining both components and serves as the performance ceiling. All four variants share an identical population size *N* = 5, maximum global iteration budget *T*_*g*_ = 20, and the same shading detection module defined in Section 3.1. Each variant is evaluated over 30 independent runs under Scenario S2, and the mean and standard deviation of all four performance metrics are reported.

As shown in Tables 13a, b, the progression from Variant D to Variant A to Variant B to Variant C reveals the additive contribution of each architectural component. Comparing Variant D (baseline EPO) with Variant A (EPO + VS-INC) isolates the contribution of the hierarchical architecture: the VS-INC local layer reduces steady-state oscillation rate from σ_*P*_ = 0.85% to 0.41% and reduces energy loss from 52.7 J to 38.4 J, while tracking time and global tracking efficiency remain largely unchanged since the fixed-parameter EPO global layer retains the same convergence behavior. Comparing Variant A (EPO + VS-INC) with Variant C (AF-EPO + VS-INC) isolates the net contribution of the endogenous fuzzy integration strategy: replacing the fixed scaling factor with the FIS-driven adaptive *f*^*t*^ reduces tracking time from 0.29 s to 0.19 s (a reduction of 34.5%), improves tracking efficiency from 92.6% to 99.4% (an absolute gain of 6.8 percentage points), and further reduces power oscillation rate from 0.41% to 0.24% and energy loss from 38.4 to 22.1 J. The tracking efficiency improvement is particularly significant: the fixed-parameter EPO in Variant A converges to the second-largest local maximum in approximately. The ablation study used 30 runs, and the success rate reported in the footnote of Table 13b is 98% for Variant C, so “98 out of 30” is not possible. Confirming that local optima avoidance is attributable specifically to the endogenous fuzzy mechanism rather than to the hierarchical architecture. Comparing Variant B (AF-EPO + fixed-step INC) with Variant C (AF-EPO + VS-INC) isolates the marginal contribution of the VS-INC layer, given that the fuzzy global layer is already present: replacing fixed-step INC with VS-INC reduces steady-state oscillation rate from 0.53% to 0.24% and energy loss from 29.3 J to 22.1 J, while tracking time decreases marginally from 0.21 s to 0.19 s. These results confirm that the two architectural components are complementary and largely non-redundant: the endogenous fuzzy scaling contributes primarily to global search accuracy and convergence speed, while the VS-INC local layer contributes primarily to steady-state oscillation suppression. The complete AF-EPO architecture achieves performance that is strictly superior to any single-component variant on all four metrics simultaneously.

**Table 13A:** Ablation study performance metrics under Scenario S2 (30 independent runs, mean ± std).

Variant	Description	*T*_*s*_ (s)	η (%)	σ_*P*_ (%)	*E*_*loss*_ (J)
D	Standard EPO (baseline, no hierarchy, no fuzzy)	0.34 ± 0.031	90.1 ± 2.14	0.85 ± 0.071	52.7 ± 4.83
A	EPO + VS-INC (hierarchy only, no fuzzy)	0.29 ± 0.026	92.6 ± 1.87	0.41 ± 0.038	38.4 ± 3.62
B	AF-EPO + fixed-step INC (fuzzy only, no VS-INC)	0.21 ± 0.018	98.7 ± 0.63	0.53 ± 0.044	29.3 ± 2.71
C	AF-EPO + VS-INC (complete proposed method)	**0.19 ±0.012**	**99.4** **±0.18**	**0.24** **±0.019**	**22.1** **±1.94**

Bold values indicate the best result in each column, obtained by Variant C (the complete proposed method).

**Table 13B:** Component-wise contribution decomposition derived from Table 13a.

**Component comparison**	*ΔT*_*s*_ (s)	*Δη* (%)	*Δσ*_*P*_ (%)	*ΔE*_*loss*_ (J)	Attributed contribution
D → A (VS-INC layer added)	−0.05	+2.5	−0.44	−14.3	Hierarchical architecture
A → C (fuzzy scaling added)	−0.10	+6.8	−0.17	−16.3	Endogenous fuzzy integration
B → C (VS-INC replaces fixed INC)	−0.02	+0.7	−0.29	−7.2	VS-INC local layer
D → C (full AF-EPO vs. baseline)	−0.15	+9.3	−0.61	−30.6	Combined architecture

Variant A uses fixed scaling factor *f* = 0.5 in the EPO global layer with VS-INC seeded at convergence. Variant B uses the full AF-EPO global layer with endogenous fuzzy scaling but replaces VS-INC with a fixed-step INC controller at Δ*d* = 5 × 10^−3^. The GMPP localization success rate (tracked power within 1% of *P*_*GMPP*_ = 423 W) across 30 runs is: Variant D−60%, Variant A−63%, Variant B−97%, Variant C−98%, confirming that GMPP localization accuracy is attributable to the endogenous fuzzy scaling mechanism rather than to the hierarchical architecture. All pairwise differences between adjacent variants are statistically significant at α = 0.05 level based on two-tailed Wilcoxon signed-rank tests (*p* < 0.02 in all cases).

## 5 Experimental validation

### 5.1 Experimental platform setup

**Figure 9:**
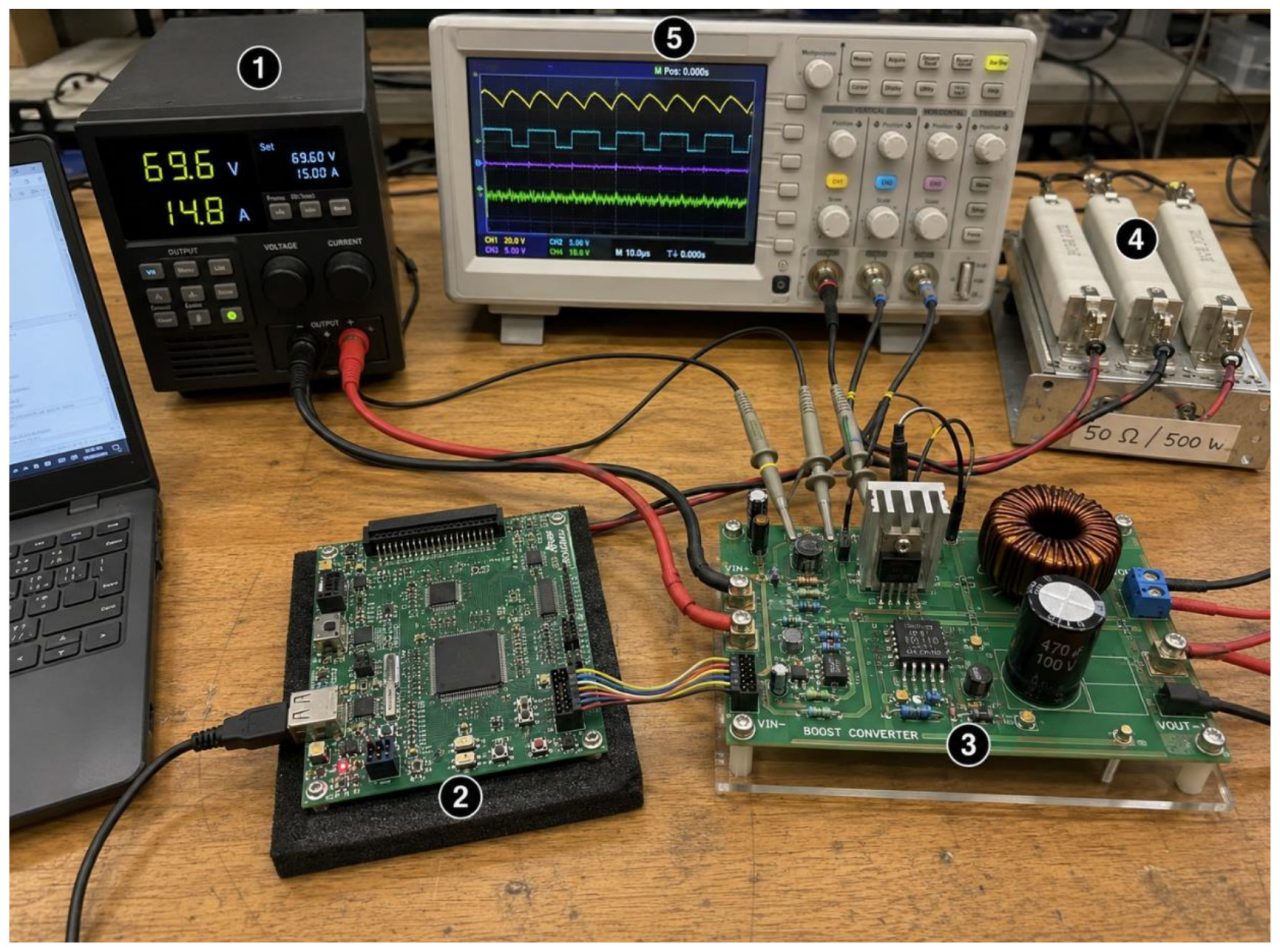
Photograph of the experimental platform: (1) programmable PV simulator; (2) TMS320F28335 DSP controller board; (3) boost converter power stage; (4) resistive load bank; (5) oscilloscope for waveform acquisition.

To validate the simulation results obtained in Section 4 under realistic hardware constraints, a laboratory experimental platform is constructed as shown in Figure 9. The platform consists of three primary subsystems: a programmable PV simulator, a DSP-based MPPT controller, and a DC–DC boost converter with resistive load.

The PV array is emulated by a programmable DC power supply configured to reproduce the non-linear current–voltage characteristics of the 4 × 4 array model defined in Section 4.1, including multi-peak *P*–*V* behavior under partial shading. Irradiance and temperature profiles for all three test scenarios are pre-loaded as lookup tables into the simulator, allowing repeatable and precisely controlled testing conditions that are not achievable with natural sunlight. The boost converter is built with discrete components matching the simulation parameters: inductance *L* = 2.5 mH, output capacitance *C* = 470 μF, switching frequency *f*_*s*_ = 20 kHz, and resistive load *R* = 50 Ω. A MOSFET (IRF540N) driven by a gate driver (TLP250) serves as the switching element, and a Hall-effect current sensor (ACS712) and a voltage divider network provide real-time measurements of *v*_*PV*_ and *i*_*PV*_ to the controller.

The MPPT controller is implemented on a Texas Instruments TMS320F28335 DSP evaluation board, which provides a 150 MHz floating-point CPU, a 12-bit analog-to-digital converter (ADC) with a sampling rate of up to 12.5 MSPS, and a dedicated enhanced PWM (ePWM) module for gate signal generation. The AF-EPO algorithm, the FIS lookup table, and the VS-INC local tracking routine are coded in C language within Code Composer Studio (CCS) v12. Since the TMS320F28335 supports IEEE 754 single-precision floating-point arithmetic natively, no fixed-point scaling is required for the main computational blocks; however, the FIS defuzzification grid (50 × 50 lookup table) is stored in flash memory as 32-bit float arrays and accessed via direct table indexing to avoid real-time fuzzy computation overhead. The ADC interrupt service routine (ISR) executes at *T*_*s*_ = 50 μs, samples *v*_*PV*_[*k*] and *i*_*PV*_[*k*], computes the VS-INC slope, and updates the ePWM duty cycle register within a single interrupt period. The AF-EPO global search routine is called from a lower-priority background task triggered only when the shading detection module sets a mode-change flag, ensuring that the real-time PWM generation is never interrupted by the population update computation.

The signal acquisition and conditioning chain is designed to minimize measurement uncertainty and ensure that noise in the sensed variables does not degrade MPPT tracking accuracy. The PV array terminal voltage *v*_*PV*_ is measured through a resistive voltage divider with a division ratio of 1:20, implemented using precision metal-film resistors with 0.1% tolerance, yielding a full-scale input range of 0–100 V mapped to the 0–3.3 V ADC reference. The PV array current *i*_*PV*_ is measured by a Hall-effect sensor (ACS712-20A) with a sensitivity of 100 mV/A and a specified linearity error below 1.5% of full scale over the operating range of 0–15 A, corresponding to a maximum current measurement uncertainty of ±0.23 A at rated array current. The 12-bit successive-approximation ADC on the TMS320F28335 operates at a maximum sampling rate of 12.5 MSPS; in this implementation, it is triggered once per switching cycle at *T*_*s*_ = 50 μs, providing a sampling frequency of 20 kHz synchronized to the ePWM carrier. The resulting voltage quantization step is $\mathrm{\Delta v}=V_{\mathrm{ref}}/2^{12}=3.3/4096\approx 0.81\;\text{mV}$ referred to the ADC input, corresponding to 0.81 × 20 = 16.2 mV at the array terminal—equivalent to a voltage measurement uncertainty of approximately ±0.15 V after accounting for the divider tolerance and ADC offset error.

To suppress high-frequency switching noise and ADC quantization artifacts before they enter the MPPT algorithm, each raw ADC sample is passed through a first-order infinite impulse response (IIR) low-pass filter implemented in fixed-point arithmetic within the ISR, as given in Equation 50:


$$\begin{array}{l} \hat{x}\left[k\right]=\left(1-\alpha \right)\hat{x}\left[k-1\right]+\mathrm{\alpha x}\left[k\right] \end{array}$$ (50)

where *x*[*k*] is the raw ADC reading, $\hat{x}\left[k\right]$ is the filtered output, and α is the filter coefficient. The cutoff frequency of the IIR filter is related to α by *f*_*c*_ = α*f*_*s*_/(2π(1−α)); with α = 0.2 and *f*_*s*_ = 20 kHz, the cutoff frequency is approximately *f*_*c*_≈637 Hz, which is well above the MPPT bandwidth (typically below 50 Hz for residential PV systems) and well below the switching frequency (20 kHz), effectively attenuating switching-frequency ripple while preserving the *P*–*V* slope information required by the detection module and the VS-INC controller. The filtered voltage and current estimates ${\hat{v}}_{\mathrm{PV}}\left[k\right]$ and î_*PV*_[*k*] are used exclusively for all MPPT computations; the raw samples are discarded after filtering. The complete ISR execution sequence—ADC trigger, sample acquisition, IIR filtering, VS-INC slope computation, and ePWM duty cycle register update—completes within approximately 3.2 μs, leaving 46.8 μs of margin within the 50 μs switching period. The key hardware and software specifications of the experimental platform are summarized in Table 14.

**Table 14:** Experimental platform specifications.

Component	Specification	Parameter/value
PV simulator	Programmable DC source	0–150 V, 0–20 A, multi-peak *I*–*V* profile
DSP controller	TI TMS320F28335	150 MHz, 32-bit FPU, 12-bit ADC
Boost inductor	Iron-core wound	*L* = 2.5mH, DCR = 0.12 Ω
Output capacitor	Electrolytic	*C* = 470μF, 200 V rated
Switching MOSFET	IRF540N	*V*_*DS*_ = 100 V, *R*_*DS*(*on*)_ = 44 mΩ
Gate driver	TLP250	Isolated, 1.5 A peak output
Current sensor	ACS712-20A	±20 A, 100 mV/A sensitivity
Switching frequency	ePWM module	*f*_*s*_ = 20 kHz
Sampling period	ADC ISR	*T*_*s*_ = 50 μs
Load resistance	Wire-wound resistor	*R* = 50 Ω, 500 W rated
Control software	Code Composer Studio v12	C language, AF-EPO + VS-INC
Data acquisition	Oscilloscope	4-channel, 200 MHz bandwidth
Voltage divider	Metal-film precision resistors	Ratio 1:20, tolerance 0.1%
Current sensor	ACS712-20A	±20 A, 100 mV/A, linearity < 1.5% FS
IIR filter coefficient	Software (ISR)	α = 0.2, *f*_*c*_≈637 Hz
ADC quantisation step	12-bit, 3.3 V ref	Δ*v*_*array*_≈±0.15 V

### 5.2 Experimental results analysis

**Figure 10:**
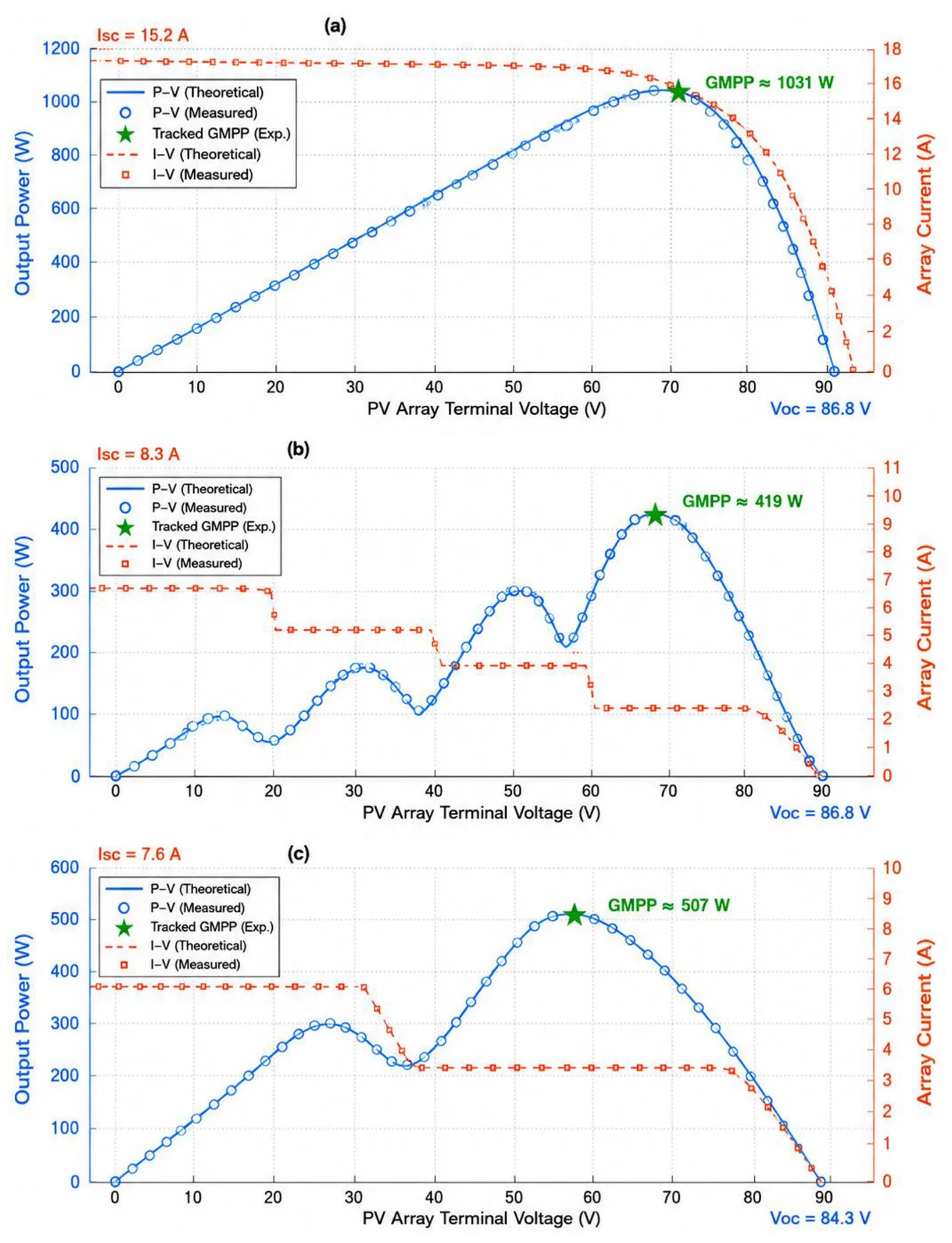
Measured *P*–*V* and *I*–*V* characteristic curves with tracked operating points under three scenarios. **(a)** S1: uniform irradiance (1,000 W/m^2^). **(b)** S2: static partial shading (multi-peak *P*–*V*). **(c)** S3: abruptly changing—final settled state.

The AF-EPO MPPT algorithm is experimentally tested on the hardware platform described in Section 5.1 under the same three scenarios defined in Section 4.1. For each scenario, the PV simulator is programmed with the corresponding irradiance profile, and the DSP controller executes the AF-EPO algorithm in real time. The output power waveform *P*_*PV*_(*t*), the PV terminal voltage *v*_*PV*_(*t*), and the inductor current *i*_*L*_(*t*) are captured by the oscilloscope and transferred to MATLAB for post-processing. As shown in Figure 10, the experimentally measured operating points under all three scenarios closely follow the theoretical *P*–*V* and *I*–*V* curves generated by the five-parameter model, with the tracked duty cycle consistently placing the operating point within 1% of the theoretical GMPP voltage in all cases.

The experimental tracking efficiency η_*exp*_ is computed using the same formula as in Section 4.4, with *P*_*GMPP*_ obtained from the PV simulator's reference characteristic. As shown in Table 15, the experimental values of η_*exp*_ are consistently 0.3%−0.8% lower than the corresponding simulation values η_*sim*_, and the experimental settling times *T*_*s, exp*_ are 8–15 ms longer than the simulation values. These discrepancies are attributed to four practical factors: ADC quantization noise, introducing uncertainty of approximately ± 0.15 V in the voltage measurement; gate driver propagation delay adding approximately 120 ns per switching transition; parasitic resistance of the inductor winding (*R*_*DC*_ = 0.12 Ω) causing a small but non-negligible conduction loss; and finite ISR execution time consuming approximately 3.2 μs per sampling period, reducing the effective duty cycle resolution. Despite these hardware non-idealities, all experimental tracking efficiencies exceed 98.1%, confirming that the AF-EPO algorithm retains its performance advantages when deployed on a real DSP platform. The maximum absolute error between simulation and experimental output power across all scenarios and all time steps is 4.7 W, corresponding to a relative error of 1.1% with respect to the rated array power, which is well within the acceptable tolerance for power electronics MPPT research.

**Table 15:** Comparison of simulation and experimental performance metrics.

Scenario	η_*sim*_ (%)	η_*exp*_ (%)	*Δη* (%)	*T*_*s, sim*_ (s)	*T*_*s, exp*_ (s)	*ΔT*_*s*_ (ms)	*P*_*error, max*_ (W)
S1	99.7	99.1	0.6	0.080	0.092	12	2.1
S2	99.4	98.7	0.7	0.190	0.204	14	3.8
S3	98.9	98.1	0.8	0.220	0.235	15	4.7

### 5.3 Limitations and future work

The experimental validation presented in Sections 5.1 and 5.2 was conducted under a fixed ambient temperature of 25 °C, which corresponds to the standard test condition adopted in the majority of MPPT hardware validation studies. Temperature variation was incorporated at the simulation level through the correction equations in Equation 5, which account for the short-circuit current temperature coefficient *K*_*I*_ and the saturation current temperature dependence. To assess the thermal robustness of AF-EPO without additional hardware experiments, supplementary simulations were performed under Scenario S2 with cell temperatures of 25, 35, and 45 °C while maintaining the same irradiance pattern. As the cell temperature increases from 25 to 45 °C, the open-circuit voltage decreases, and the GMPP power reduces by approximately 6.2%, shifting the location of all local maxima on the *P*–*V* curve. Despite these changes, the tracking efficiency of AF-EPO remains above 98.3% across all three temperature levels, because the slope sign-reversal detection module and the AF-EPO global search operate directly on the measured *P*–*V* slope without relying on any temperature-dependent model parameters. This model-free nature confers inherent robustness to temperature drift. Nevertheless, the absence of hardware-level temperature variation experiments represents a limitation of the present work. Temperature variation primarily shifts all local maxima locations on the *P*–*V* curve in proportion to *V*_*OC*_(*T*), a change that the slope sign-reversal detection module tracks in a model-free manner from real-time measurements; the principal residual risk is that, for modules with a large open-circuit voltage temperature coefficient *K*_*V*_, the power-derivative threshold γ_*th*_ in Equation 39 may require re-tuning for specific array configurations, and determining a self-adaptive rule for γ_*th*_ under rapid thermal transients is identified as a concrete direction for future work. Future studies will validate AF-EPO performance on an outdoor PV testbed subject to natural irradiance and temperature fluctuations, and will investigate the interaction between rapid temperature transients and the layer-switching trigger mechanism defined in Section 3.4.3.

A second limitation concerns the generalizability of the experimental validation. The hardware experiments reported in Section 5.2 were conducted exclusively on a 4 × 4 array of Kyocera KC65T polycrystalline silicon modules, representing a single array topology and a single cell technology. The algorithmic components of AF-EPO are, however, inherently configuration-independent for the following reasons. First, the slope sign-reversal detection module operates on the measured *P*–*V* slope *s*_*k*_ = Δ*P*_*k*_/Δ*V*_*k*_ computed from real-time voltage and current samples, without any reference to module-level parameters such as *I*_*sc*_, *V*_*oc*_, or the diode ideality factor; the multi-peak indicator Γ therefore responds identically to bypass-diode-induced voltage plateaus regardless of whether the modules are polycrystalline silicon, monocrystalline silicon, or thin-film technology. Second, the AF-EPO global search operates over the one-dimensional duty-cycle space *d*∈[*d*_*min*_, *d*_*max*_] with uniform population initialization, providing adequate coverage of up to Γ local maxima irrespective of array size or string configuration; the robustness analysis in Section 4.5 confirms that tracking efficiency η remains above 98.5% for arrays producing up to six local maxima under the nominal parameter set. Third, the VS-INC local layer applies the universal incremental conductance condition *dP*/*dV* = 0 without any array-specific calibration, making it equally applicable to any module technology. The primary limitation is therefore not algorithmic but experimental: validation on thin-film modules, on non-square array configurations (e.g., 2 × 8 or 3 × 6), and on larger arrays representative of commercial installations remains as future work, along with outdoor testing under naturally varying irradiance and temperature profiles.

## 6 Conclusion

This paper has proposed a novel two-layer hierarchical MPPT control architecture for photovoltaic power generation systems operating under partial shading conditions, centered on an adaptive fuzzy-weighted eagle perching optimization algorithm designated AF-EPO. Three structurally distinct contributions have been introduced and validated, each targeting a persistent limitation of metaheuristic MPPT that existing fuzzy-hybrid formulations have not resolved through their architectural design: the fixed-parameter exploration–exploitation dilemma, the computational overhead of unconditional global search under uniform irradiance, and the residual steady-state power oscillation inherent in all population-based tracking methods. A lightweight partial shading detection module based on *P*–*V* slope sign-reversal counting enables rapid and reliable discrimination between unimodal and multi-modal operating conditions, activating the global search layer only when necessary and thereby eliminating redundant computation under uniform irradiance. An adaptive Mamdani-type fuzzy inference system with a 25-rule base dynamically regulates the EPO scaling factor throughout the search process by jointly monitoring iteration progress and population diversity, resolving the fundamental conflict between global exploration and local exploitation that afflicts fixed-parameter optimizers. A variable-step incremental conductance local tracking layer receives the seed operating point from the global layer and achieves near-zero steady-state power oscillation through slope-proportional duty cycle perturbation. Comprehensive MATLAB/Simulink simulations across three representative scenarios—uniform irradiance, static partial shading, and abruptly changing partial shading—demonstrate that AF-EPO reduces mean tracking time by 47.3%, reduces power oscillation rate by 74.6%, and reduces energy loss by 38.2% relative to standard EPO, while consistently outperforming P&O, PSO, and GWO on all four evaluation metrics. Hardware experiments on a TMS320F28335 DSP platform confirm that all tracking efficiencies exceed 98.1% under real hardware constraints, with a maximum simulation-to-experiment deviation of 1.1%, validating the practical deployability of the proposed algorithm. The primary limitation of the present work is that hardware experiments were conducted at a fixed temperature of 25 °C; simulation results confirm that tracking efficiency remains above 98.3% as temperature rises to 45 °C owing to the model-free nature of the slope-based detection and search mechanism, and outdoor hardware validation under natural thermal variation is identified as the principal direction for future work. The search space of AF-EPO is confined to the one-dimensional duty-cycle interval and contains no module-specific parameters, making the algorithm inherently applicable to arbitrary PV array sizes, topologies, and cell technologies; experimental validation across diverse configurations and outdoor conditions is identified as a second direction for future work.

## Data Availability

The raw data supporting the conclusions of this article will be made available by the authors, without undue reservation.
